# Microfluidics and Nanofluidics in Strong Light–Matter Coupling Systems

**DOI:** 10.3390/nano14181520

**Published:** 2024-09-19

**Authors:** Evelyn Granizo, Irina Kriukova, Pedro Escudero-Villa, Pavel Samokhvalov, Igor Nabiev

**Affiliations:** 1 Life Improvement by Future Technologies (LIFT) Center, 143025 Moscow, Russia; aleroman16@hotmail.com (E.G.); irina.kryukova.mephi@gmail.com (I.K.); p.samokhvalov@gmail.com (P.S.); 2 Laboratory of Nano-Bioengineering, Moscow Engineering Physics Institute, National Research Nuclear University MEPhI, 115409 Moscow, Russia; 3 Facultad de Ingeniería, Universidad Nacional de Chimborazo, Riobamba 060108, Ecuador; pedro.escudero@unach.edu.ec; 4 Department of Clinical Immunology and Allergology, Institute of Molecular Medicine, Sechenov First Moscow State Medical University (Sechenov University), 119146 Moscow, Russia; 5 BioSpectroscopie Translationnelle (BioSpecT)—UR 7506, Université de Reims Champagne-Ardenne, 51100 Reims, France

**Keywords:** strong light–matter coupling, microfluidics, nanofluidics, polaritons

## Abstract

The combination of micro- or nanofluidics and strong light–matter coupling has gained much interest in the past decade, which has led to the development of advanced systems and devices with numerous potential applications in different fields, such as chemistry, biosensing, and material science. Strong light–matter coupling is achieved by placing a dipole (e.g., an atom or a molecule) into a confined electromagnetic field, with molecular transitions being in resonance with the field and the coupling strength exceeding the average dissipation rate. Despite intense research and encouraging results in this field, some challenges still need to be overcome, related to the fabrication of nano- and microscale optical cavities, stability, scaling up and production, sensitivity, signal-to-noise ratio, and real-time control and monitoring. The goal of this paper is to summarize recent developments in micro- and nanofluidic systems employing strong light–matter coupling. An overview of various methods and techniques used to achieve strong light–matter coupling in micro- or nanofluidic systems is presented, preceded by a brief outline of the fundamentals of strong light–matter coupling and optofluidics operating in the strong coupling regime. The potential applications of these integrated systems in sensing, optofluidics, and quantum technologies are explored. The challenges and prospects in this rapidly developing field are discussed.

## 1. Introduction

**Figure 1:**
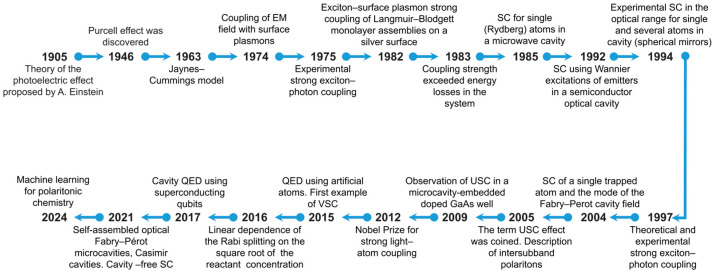
Strong light–matter coupling (SC) timeline: from cavity quantum electrodynamics (QED) to ultrastrong coupling (USC). The data are from Refs. [1,2,5,6,7,8,9,10,11,12,13,14,15,16,17,18,19,20,21,22,23,24,25,26].

In 1905, Einstein proposed an explanation of the photoelectric effect [1] assuming that light is made up of particles, which suggested the existence of light energy quanta now called photons. This wave–particle duality of light became a fundamental concept in quantum theory, playing a pivotal role in the advancement of photonics. A great leap in this field was the discovery of the Purcell effect in 1946 [2], which substantially advanced our understanding of how light interacts with matter. Since then, light–matter interactions have been intensely studied, with considerable advances made in theoretical simulation, experimental research, and practical applications. The development of cavity quantum electrodynamic (CQED) systems has been one of the major advances in this field. These systems consist of optical cavities containing one or several atoms, organic molecules, or artificial atoms, such as quantum dots (QDs). These and other advances mark the evolution of the strong light–matter coupling (SC) systems, which has led to a number of important applications in the fields of nanophotonic devices, manipulation of quantum systems, high-precision measurements [3], and quantum information processing (QIP) [4]. The timeline of research and developments in strong light–matter coupling systems is shown in Figure 1.

Some of the most outstanding advances of the last years are related to SC in a liquid environment. Integrating strong light–matter coupling systems with micro-/nanofluidics offers immense promise for advancing numerous application fields, including quantum optics, biosensing, and chemical processing. These hybrid systems enable precise manipulation of molecules and photons in fluidic environments, opening new pathways for controlling light–matter interactions on the micro- and nanoscales. The dynamic and tunable nature of fluidic environments allows researchers to manipulate light–matter interactions more effectively, particularly by providing real-time control of molecular interactions and photonic properties. This offers new possibilities for designing advanced devices that can increase reaction yields, improve sensing accuracy, and scale up the overall efficiency of light-driven processes.

Some of the advantages of combining SC and micro-/nanofluidics are a reduced consumption of expensive materials and reagents, a shortened reaction time and an increased product yield in chemistry-related fields, an improved sensitivity in sensing applications, and portability. Researchers have succeeded in developing a variety of micro- and nanofluidic techniques and technologies using plasmon nanostructures [27,28], whispering gallery resonators [29,30,31], and photonic crystals (PCs) [32]. However, despite the significant potential, there are substantial gaps and challenges that remain unaddressed in this emerging field. One major challenge is the fabrication of nano- and microscale optical cavities and their integration into fluidic systems where maintaining precise control and stability over long time periods is particularly difficult. Furthermore, the strong coupling regime requires both the optical cavity and the fluidic environment to be carefully engineered to maximize the interaction strength, often requiring complex and delicate setups. Another key issue is scalability, because while these systems have demonstrated exciting results on a small scale, their application in larger or more practical systems is still limited by fabrication constraints and stability concerns. Additionally, achieving high sensitivity and signal-to-noise ratio remains a crucial hurdle, especially in real-time monitoring and control of light–matter interactions in fluidic environments. The inherent instability of flowing fluids and the challenge of maintaining consistent optical properties further complicate the development of reliable, reproducible devices.

Our review addresses these issues by summarizing recent research and critically analyzing the emerging trends in integrating micro- and nanofluidics with strong light–matter coupling.

We aim to elucidate the fundamental principles behind these systems, experimental techniques used to study light–matter coupling in them, and technological implications of the observed phenomena. The insights presented in this review not only clarify the current state of research but also inspire future trends and innovations in this field.

## 2. Fundamentals of Strong Light–Matter Coupling

### 2.1. Light–Matter Coupling Regimes

**Figure 2:**
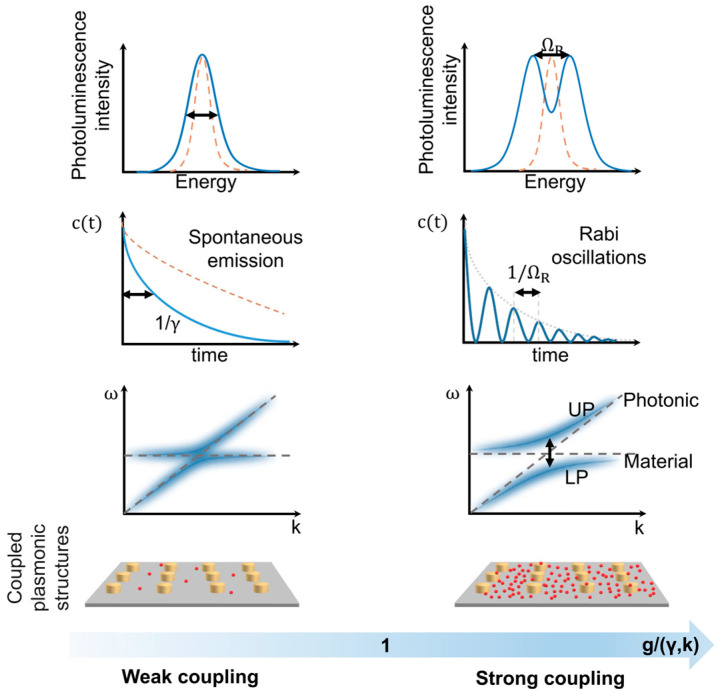
Schematics of weak (on the **left**) and strong (on the **right**) light–matter coupling. Here, a plasmonic resonator is represented by an array of periodically arranged gold rings coupled with emitters. In the weak coupling regime, the emitter decay rate c(t) is enhanced (Purcell enhancement) without a substantial modification of the emitter eigenstate. In the strong coupling regime, the emitter decays at a rate governed by the Rabi frequency $\Omega_{R}$, which leads to the formation of two new eigenstates, exhibiting anti-crossing behavior: upper and lower polaritons (UP and LP, respectively).

When a two-level system (e.g., an atom or a molecule) has a transition that is resonant with the confined electromagnetic (EM) field, two regimes of light–matter interaction could be distinguished: weak coupling and strong coupling. The light–matter coupling strength (g) is then compared with two parameters: the photon decay rate of the cavity (κ) and the nonresonant decay rate of the molecule (γ) [13] (Figure 2).

When g<<(κ,γ), the system is considered to be in a weak coupling regime. This means that the exchange rate is smaller than the loss rates; hence, the excitation is lost before it can be shared between the two-level system and the cavity. The weak coupling regime is related to the Purcell enhancement of spontaneous emission (F) [33]:

(1) $$F=\frac{3Q\lambda^{3}}{4\pi^{2}Vn^{3}},$$

where Q is the quality factor, V is the mode volume, λ is the vacuum wavelength, and n is the refractive index (RI) of the microcavity. As an example, this effect predominates when the molecule is placed next to a metal surface or nanostructure [34], contributing to the tuning of the photophysics and photochemistry (i.e., plasmon chemistry) of the system, which can be used for controlling chemical reactions.

When g>>(κ,γ), the system is in a strong coupling regime. This means that the coupling is strong enough to overcome the dissipation. Therefore, it is characterized by a reversible coherent exchange of energy (known as Rabi oscillation) between the field in the cavity and the electronic or vibrational states of the embedded matter, leading to the formation of two polaritonic modes (P^+^ and P^−^), which emerge as two distinct peaks in the energy spectrum of the system. In this regime, the light–matter interaction cannot be treated perturbatively and requires theoretical methods to describe the interaction between photons, electrons, and nuclei on different length and time scales.

Initially, in the uncoupled state, the transition energy of the two-level system is constant and has no dispersion, while the energy of the cavity eigenmode exhibits angular dispersion and its minimum coincides with the transition energy at resonance. In the strong coupling regime, both hybridized states exhibit dispersion. It is important to note that the minimum of the cavity energy increases, resulting in a gap between the hybridized states observed at any angle. This effect is termed anti-crossing because even under resonance conditions the two branches of the hybridized states diverge. The anti-crossing of the dispersion curves of polariton states is the most reliable criterion of the strong coupling regime in the system.

On the basis of the Jaynes–Cummings model, in the absence of dissipation, the Rabi splitting is

(2) $$\hbar \Omega_{R}=2d\sqrt{\frac{\hbar \omega }{2\varepsilon_{0}V}}\sqrt{n_{ph}+1},$$

where ℏω is the cavity resonance or molecular transition energy, $\varepsilon_{0}$ is the vacuum permittivity, V is the mode volume, d is the transition dipole moment of the molecule, and $n_{ph}$ is the number of photons involved in the coupling. Rabi splitting can also occur in the absence of photons in the cavity when $n_{ph}=0$. This phenomenon is determined by the interaction of matter with zero-point EM fluctuations with the energy of ℏω/2 and is known as vacuum field Rabi splitting [35].

In addition, a strong coupling regime is substantially facilitated by the so-called collective coupling, where a large number of emitters, e.g., molecules, couple to one optical mode. In this case, Rabi splitting is transformed as follows:

(3) $$\hbar \Omega_{R}=2d\sqrt{N}\sqrt{\frac{\hbar \omega }{2\varepsilon_{0}V}},$$

This expression shows that a small mode volume and a considerable amount of molecules with a large transition dipole moment are required to confine the EM field and increase the coupling strength g and, therefore, the value of the Rabi splitting. Owing to the collective effects of an assemble of vibrational modes, a strong coupling regime can be achieved even at room temperature [20,36,37]. The interaction of the EM field and an assemble of N molecules results in the formation of N + 1 polariton states. However, only two of them can be observed experimentally, e.g., as two peaks in the absorption spectrum, and, therefore, are termed bright states. The other N − 1 collective states cannot be directly excited with light and, hence, cannot be observed, so they are termed dark states [38]. Thus, the situation remains similar to the case of a single two-level system in terms of observable energy states.

In addition, intermolecular interactions are important to consider in collective coupling. If the distance between molecules is small enough, Coulomb interactions between the transition dipole moments of different molecules become significant. Therefore, the transfer of excitation energy from one molecule to another is facilitated; i.e., there is delocalization of the excitation. Consequently, the transfer processes that favor delocalization, are excellent candidates for potential enhancements under strong light–matter coupling [39,40].

In the strong coupling regime, either an electronic or a vibrational transition can be strongly coupled to the optical cavity mode, termed electronic strong coupling (ESC) and vibrational strong coupling (VSC), respectively. VSC is typically easier to obtain because vibrational modes are in the infrared range, thus requiring cavity lengths of the order of micrometers. VSC has been demonstrated to considerably modify the chemical reactivity of molecules, either enhancing or reducing it [22,41,42,43,44]. The formation of the hybrid states in the VSC regime results in the so-called vibro-polaritonic (VP) modes. ESC occurs in the visible range, which requires cavities smaller in size, making their engineering a real challenge. In the case of ESC, various properties and processes have been found to be altered, such as work function, charge transport, energy transfer, intersystem crossing, and nonlinear optics, along with chemical reactivity.

**Figure 3:**
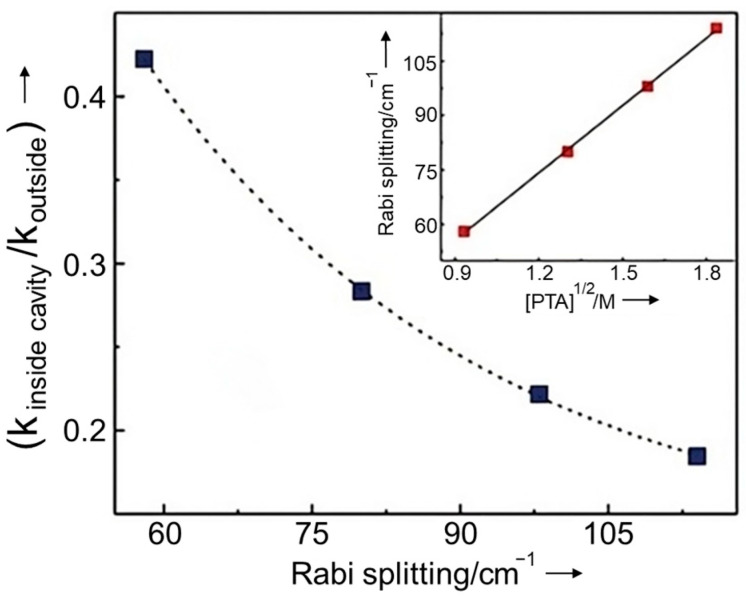
The decrease in the ratio of the reaction rates under vibrational strong coupling and outside the cavity as a function of the Rabi splitting energy. The inset shows the linear dependence of the Rabi splitting on the square root of the 1-phenyl-2-trimethylsilylacetylene (PTA) concentration. Adapted with permission from Thomas et al. (2016) [22], published by Wiley-VCH GmbH & Co. KGaA, Hoboken, NJ, USA.

The strong coupling regime is not always feasible due to the weak oscillator strength of the vibration to be coupled or the restricted solubility of the active molecules in the solvent used. In this case, the active solute molecules can be dissolved in a solvent or a matrix that has a strong vibrational band interacting and matching with the vibrational mode of the solute. This is called cooperative coupling [44,45]. In cooperative coupling, the reactants indirectly couple with the confined EM field through their match with the vibrational modes of media. If the solvent is involved in VSC, all the solvent molecules surrounding the solute are vibrating in phase, which, in turn, drives and couples the solute vibration. As the vibrational energy of a chemical bond is changed by VSC, the ground potential energy surface of the chemical reactions involving this bond is perturbed. In 2016, it was demonstrated [22] that the relative change in the reaction rate in the VSC regime depends on the Rabi splitting energy, which is proportional to the square root of the concentration of the reactant (C) (Figure 3):

(4) $$\hbar \Omega_{R}\propto \sqrt{\frac{N}{V}}=\sqrt{C},$$

where N and V are the number of emitters and the cavity volume, respectively.

### 2.2. Optical Characteristics

The confinement efficiency under a strong light–matter coupling regime is typically described in terms of the quality factor (Q-factor), photon lifetime (τ), and mode volume (V). The Q-factor measures the resonant quality or the capacity for confining light inside the microcavity and can be calculated as follows:

(5) $$Q=\frac{\lambda_{cf}}{\Delta \lambda },$$

where $\lambda_{cf}$ is the central wavelength of the resonance mode, and Δλ is its spectral linewidth, commonly termed the full width at half maximum (FWHM). Microcavities with Q-factors in the range from 10^3^ to 10^6^ are referred to as high-Q cavities, and those with Q-factor higher than 10^7^, as ultrahigh-Q cavities [46].

The photon lifetime is defined as the time that a photon is trapped inside the microcavity before being dissipated and it is proportional to the Q-factor. Hence, fabrication of structures with a high Q-factor is critically important. The photon lifetime can be calculated as follows:

(6) $$\tau =\frac{Q}{\omega_{cf}},$$

where $\omega_{cf}$ is the central frequency of the resonance mode.

**Figure 4:**
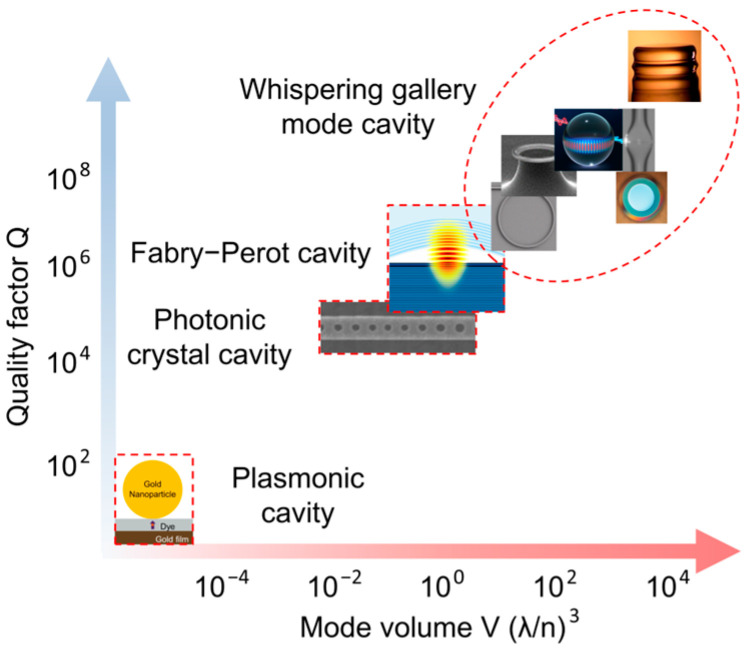
The quality factor, Q, versus mode volume of different types of cavities. Adapted with permission from Liu et al. (2022) [49], published by Elsevier, Amsterdam, The Netherlands.

The mode volume, also referred to as the effective mode volume, in contrast to the geometric volume, which is only relevant to the physical size of the cavity, describes how efficiently the cavity confines the field and is determined from the spatial distribution of the EM field in the cavity:

(7) $$V_{eff}=\frac{\int_{V}\varepsilon (r){\left|E(r)\right|}^{2}d^{3}r}{\max(\varepsilon (r){\left|E(r)\right|}^{2})},$$

where ε(r) is the permittivity at position r, E(r) is the electric field strength at position r, and V is the volume of the microcavity. This expression means that the energy density increases as the mode volume decreases. Therefore, a smaller mode volume can lead to a stronger interaction between the light and the system. The mode volume is usually expressed in units equal to the cube of the wavelength divided by the RI of the cavity material, i.e., ${\left(\lambda /n\right)}^{3}$. Since the feasible size of a dielectric cavity cannot be smaller than half the wavelength considered, the minimum available mode volume for a cavity-type resonator is limited by $V_{eff}\approx 1$ [47]. Therefore, when smaller mode volumes $V_{eff}<1$ are desired, nanoscale optical cavities, such as plasmonic cavities, are used where the diffraction limit is overcome and the mode volumes can be as small as $V_{eff}\approx 10^{-6}$. Yet, in this case the Q values are low because of high losses in the metal (Q≈10) [48]. Figure 4 shows the comparison of Q-factor versus the mode volume for different types of cavities.

**Figure 5:**
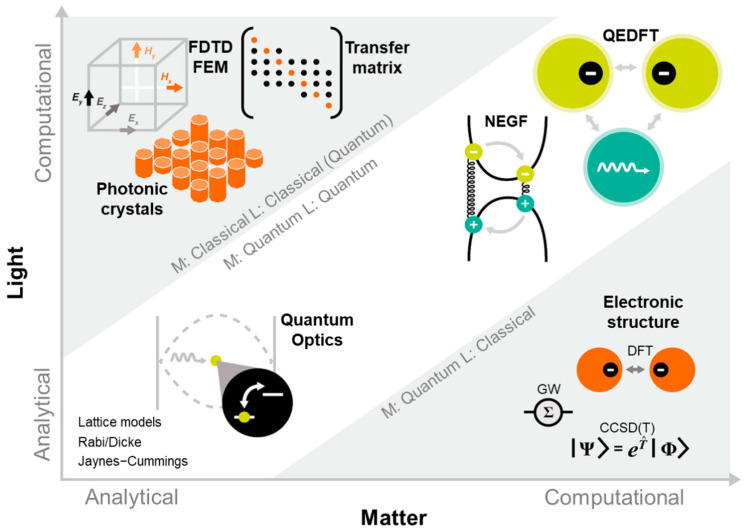
Numerical and computational methodologies for describing light–matter interactions. Adapted with permission from Flick et al. (2018) [59], published by De Gruyter, Berlin, Germany.

To describe the light matter–interaction, numerical and computational methodologies could be used, which can be categorized according to the level of theory complexity (Figure 5). From the computational approach, methods such as the finite-difference time-domain (FDTD), finite element method (FEM), and the transfer matrix approach use Maxwell’s equations for the light field. FDTD excels at simulating light propagation and scattering for time-domain analysis. This method is typically used to determine the mode volume [47,50,51] of a system in a cavity, the dispersion diagram, Q-factor, and mode pattern of the cavity. FEM excels in frequency-domain analysis, high-accuracy modeling of complex geometries, and situations requiring detailed spatial resolution [52]. The transfer matrix method is used to study the reflectivity spectra and the complex frequencies of optical eigenmodes of the structure as functions of the vacuum Rabi frequency [53]. One step further in the computational approach is the density functional theory (DFT) method that solves the electronic Schrödinger equation and often ignores the transverse electromagnetic field effects. Here, the matter and light are treated from the quantum and classical approach, respectively. To overcome the classical approximation of the electromagnetic field, the full complexity of the system must be considered by treating the matter and light on a coherent quantized basis. Therefore, quantum-electrodynamical density functional theory (QEDFT) [54], quantum electrodynamics coupled-cluster theory (QED-CC) [55] and two quantum electrodynamical coupled-cluster singles–doubles (QED-CCSD) are ab initio methodologies that accurately model correlated electron–photon systems. QEDFT has been used to describe SC; however, it requires massive computing power. These methodologies have been used recently to calculate local polaritonic effects [56], ionization energies [57], and ground state modifications [58].

The theoretical bases used to describe the SC include classical and quantum models. However, in view of the complexity of the problem, the classical interaction of the EM fields with matter is not sufficient to describe the SC. Therefore, model Hamiltonians are used in most theoretical studies, which are mostly based on the Jaynes–Cummings or Dicke model for single or multiple systems, respectively. These classical and quantum theoretical models are described in the following section.

### 2.3. Classical Approach

In the classical approach, the coupling of an optical mode and matter (quantum systems such as atoms, molecules, and solid-state systems) is described in terms of the classical EM theory using the analogy of two coupled harmonic oscillators. In an uncoupled system, the behaviors of the harmonic oscillators are independent of each other. When the coupling is strong enough, the two oscillators start to periodically transfer energy to each other, behaving as a single entity. The periodic exchange of energy is represented by a third spring with an interaction spring constant g, which corresponds to the coupling strength of the system. The classical motion description for two dissipative harmonic oscillators can be expressed as [38]

(8) $$m_{A}{\ddot{x}}_{A}=-k_{A}x_{A}+g(x_{B}-x_{A}),$$

(9) $$m_{B}{\ddot{x}}_{B}=-k_{B}x_{B}+g(x_{A}-x_{B}),$$

where $m_{i}$, $x_{i}$ and $k_{i}$ are the mass, displacement, and spring constant of the two oscillators. These equations assume that the coupling rate is much larger than the dissipation rate; i.e., the system is in a strong coupling regime. Defining $\omega =\sqrt{k/m}$ for each oscillator, we obtain two new normal modes:

(10) $$\omega_{\pm }=\frac{1}{2}\left(\omega_{A}+\omega_{B}\pm \sqrt{{\left(\omega_{A}-\omega_{B}\right)}^{2}+4\Omega^{2}}\right),$$

where $\omega_{A}$, $\omega_{B}$, and Ω are the frequencies of the oscillators *A* and *B* and the frequency splitting, respectively. When the system is at resonance, $\omega_{A}=\omega_{B}\equiv \omega$, this yields two eigenvalues $\omega_{\pm }=\omega \pm \Omega$ with two new normal modes differing from each other by 2Ω where the splitting (Ω) between the two new modes depends on the coupling strength g.

However, it should be noted that the classical approach has limitations, especially in the description of phenomena occurring at the quantum level. For a more accurate description of strong light–matter coupling, a quantum mechanical approach, such as the Jaynes–Cummings model or Dicke model, is often necessary.

### 2.4. Quantum Approach

In the quantum approach, both the atom and light are quantized. The main difference between the quantum approach and the classical description is the consideration of vacuum EM fluctuations in empty space. These vacuum fluctuations represent the optical modes with the zero-point energy (ground state energy) of the quantized EM field. In free space, the EM field has a continuum of modes that typically interact weakly with the system. EM fluctuations affect the properties of molecules and materials, such as spontaneous emission. One way to enhance this interaction is to place the system inside an optical cavity. In this confined geometry, the EM field presents a discrete spectrum of modes. In addition, the field can be much more localized than in free space. This localized EM field leads to a stronger field enhancement, which increases the emission quantum yield because it strengthens the interaction between light and matter, resulting in more efficient energy transfer due to an increase in the excitation and emission rates of the molecules [60,61].

Around 1936, Rabi [62] introduced a model to discuss the effect of a rapidly changing weak magnetic field on an oriented atom that has a nuclear spin. This model considers a two-state system consisting of a single atom (N = 1). The atom is treated as a quantum object, and the field is treated classically as a rotating field. In 1954, Dicke proposed a version for a single quantum field mode with multiple two-level atoms (N > 1). It describes the collective behavior of the atoms and the field, considering the possibility of cooperative effects, such as superradiance and subradiance. This makes it particularly useful for studying systems with many atoms where collective behavior plays an important role.

Despite its simplicity, the quantum Rabi model was not regarded as exactly solvable. To solve the model, the rotation wave approximation (RWA), in which the counter-rotating term (CRT) is neglected, was advanced by Jaynes and Cummings in 1963 [5]. The Jaynes–Cummings (JC) model describes the interaction between a single two-level atom (N = 1) and a single mode of the EM field, taking into account the quantum nature of both the atom and the field. The JC model is readily solved, including the dynamics of the EM field, making it more comprehensive than the Rabi model. The JC model has been successfully used to understand a range of experimental phenomena, such as vacuum Rabi mode splitting [12] and quantum Rabi oscillation [63]. In 1968, Tavis and Cummings proposed an extension of the JC model for the case of several (N > 1) identical noninteracting two-level systems coupled to a single-mode quantized radiation field. Thus, the Dicke model is a multi-atom version of the quantum Rabi model, whereas the Tavis–Cummings (TC) model is an extension of the JC model for N > 1. Table S1 (Supplementary Materials) summarizes the main characteristics of each model.

Let us consider the model of a single-mode field interacting with a two-level system. The full JC Hamiltonian takes the form

(11) $${\hat{H}}_{JC}=\hbar \omega_{c}{\hat{a}}^{†}\hat{a}+\frac{\hbar \omega_{a}}{2}{\hat{\sigma }}_{z}+\hbar g({\hat{\sigma }}_{+}\hat{a}+{\hat{\sigma }}_{-}{\hat{a}}^{†}),$$

where $\hbar \omega_{c}$ is the resonance of the electric field, $\hbar \omega_{a}=E_{e}-E_{g}$ represents the atomic transition energy, ${\hat{a}}^{†}$ and $\hat{a}$ are the creation and annihilation operators of a single-mode bosonic field satisfying the canonical commutation relation $[\hat{a},{\hat{a}}^{†}]=1$, ${\hat{\sigma }}_{z}=|e\rangle \langle e|-|g\rangle \langle g|$ is the Hermitian inversion operator, ${\hat{\sigma }}_{+}=|e\rangle \langle g|$ and ${\hat{\sigma }}_{-}=|g\rangle \langle e|$ are the atomic rising and lowering operators, and g is proportional to the dipole moment. The JC Hamiltonian has the following eigenstates:

(12) $$|1n\rangle =\frac{1}{\sqrt{2}}\left(|e,n\rangle +|g,n+1\rangle \right),$$

(13) $$|2n\rangle =\frac{1}{\sqrt{2}}\left(-|e,n\rangle +|g,n+1\rangle \right),$$

The new, hybridized eigenstates (12)–(13) are called *dressed* states of the atom, i.e., the eigenstates of the atom “dressed” by the cavity mode. A detailed description is presented in Section A of the Supplementary Materials.

In summary, the Rabi model is a simplified description of the light–matter interaction for a single two-level atom, with the atom treated as quantized and the field treated as a classically rotating field, whereas the JC model extrapolates this situation to a quantized EM field. The Dicke model, on the other hand, considers collective behavior of many atoms interacting with a common field. Each model is useful for different types of systems and highlights different aspects of the light–matter interaction. Research in fully quantum models is still underway to understand the effects related to strong coupling. Due to the mathematical difficulties entailed by the inclusion of the counter-rotating terms, the Rabi model has been mostly analyzed either numerically or by approximate methods appropriate for each particular situation. Connecting these predictions to experimental observations is quite challenging [64,65,66]. In the following section, we describe experimental models of strong light–matter coupling integrating micro- and nanofluidics.

## 3. Methods and Techniques for Obtaining Strong Light–Matter Coupling in Micro- and Nanofluidic Systems

Micro- and nanofluidics imply accurate control and manipulation of fluids on the microscale (1–100 μm) and nanoscale (1–100 nm), in so-called microchannels and nanochannels, respectively [67]. The numerous techniques for the fabrication of micro-/nanofluidic devices developed to date are described in [68,69]. In terms of fabrication, the earliest devices were engineered using traditional microfabrication methods, such as photolithography [70] and soft lithography [71], and were mainly used for academic research. As the field has developed, the range of methods and techniques for fabricating microfluidic devices has expanded to encompass 3D printing, laser machining, and micro-milling, enabling the design of more complex and diverse devices. Similarly, the evolution of nanofluidics has been driven by advances in nanofabrication techniques, such as electron beam lithography and focused ion beam milling, allowing precise manipulation and control of a nanoscale fluid flow. For example, the fabrication of micro- and nanointerfaces with geometrically controlled ~10 nm nanochannels on a single fused-silica substrate using the lithography and etching processes has been reported. The system has been used for obtaining a pressure-driven flow of liquids in 50 nm channels [72].

Initially, microfluidic devices were based on silicon and glass. Over time, new materials, such as polymers, paper substrates, metals, hydrogels, and composites, have been included. In the case of nanofluidics, the use of nanomaterials, such as carbon nanotubes and graphene, has opened new possibilities for controlling the flow of a fluid. In general, a simple micro-/nanofluidic device allows for the storage and handling of fluidic samples or reagents, as well as waste materials, in a single compact and disposable unit, which largely facilitates automated sample supply for sensing and minimization of the sample volume. At the dawn of microfluidics, the fluid was supplied by pumps; as the technology advanced, digital microfluidics emerged instead of a continuous flow. This technology allows for transporting microdroplets by electrowetting, avoiding various potential problems such as channel clogging, air bubble formation, and sample cross-contamination.

The applications of micro- and nanofluidics have also expanded rapidly. In particular, synergy of strong light–matter coupling and micro-/nanofluidics for controlling molecular processes and chemical behavior in a cavity environment is a subject of intense experimental and theoretical research and developments [73]. This combination brings together the advantages of fluidics, such as the capacity for controlling a low sample volume and flexibility in producing multiple channels, with the possible benefits of strong coupling in biosensing and control of chemical reactions.

Micro- and nanofluidics significantly enhance light–matter coupling by reducing sample volumes, minimizing the specimen volume, and concentrating interactions within smaller spaces. This approach is particularly advantageous when dealing with costly, toxic, or rare materials, such as quantum dots and other nanoparticles. These micro- and nanoscale systems are well suited for applications requiring high sensitivity, such as single-molecule detection. In particular, nanofluidics is a powerful tool for single-molecule manipulations and analysis because the channel sizes are comparable with molecular sizes [74]. By confining individual molecules in micro- and nanochannels, these systems enable precise interactions with the localized EM field, improving the detection of subtle changes in quantum systems. On the other hand, bulk liquid sensing often deals with large ensembles of molecules, averaging interactions over large volumes and making it difficult to isolate the effects of individual interactions. The precise control of micro- and nanofluidics is crucial for dynamic sensing and allows real-time analysis [75]. Furthermore, these platforms are highly tunable, offering fine control over experimental conditions, such as temperature, pressure, and chemical composition, which is difficult to achieve in bulk liquid systems [76]. This heightened interaction sensitivity is crucial for applications that rely on strong coupling, including biosensing, nanophotonics, and quantum optics. Thus, when integrated with optical cavities, micro- and nanofluidics further amplify the interaction strength, facilitating the observation of quantum phenomena, such as Rabi splitting.

However, studying micro- and nanofluidics is not straightforward because of the numerous phenomena occurring simultaneously at these small scales. This inherently makes micro-/nanofluidics a multidisciplinary field, encompassing chemical and biological reactions, the transport of mass, charge, momentum, and energy, as well as crystallization and molecular interactions. In selecting materials for such applications, it is essential to consider factors like reproducibility, cost-effectiveness, and the specific configurations required for SC applications, as outlined below.

## 4. Configurations Combining Strong Light–Matter Coupling with Micro- and Nanofluidics

**Figure 6:**
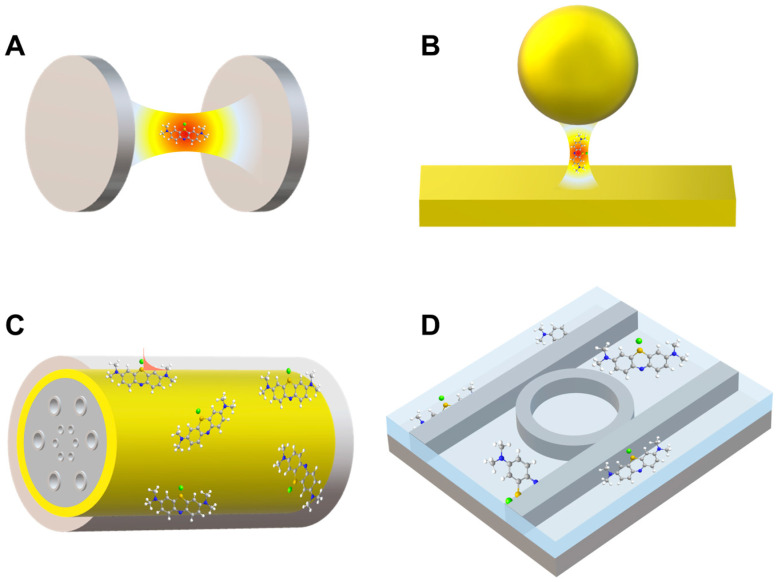
Different setups used for obtaining strong light–matter coupling for the use in micro-/nanofluidics: (**A**) Fabry–Pérot cavity, (**B**) plasmon nanocavity, (**C**) photonic crystal fiber plasmonic sensor, (**D**) whispering-gallery mode microcavity (microring resonator).

Optical microcavities, also referred to as microresonators, can be subdivided, according to their designs, into Fabry–Pérot microcavities, plasmonic nanocavities, photonic crystal microcavities, and whispering-gallery mode (WGM) microcavities (Figure 6).

### 4.1. Fabry–Pérot Microcavity

A Fabry–Pérot cavity consists of two mirrors usually separated by an adjustable spacing. The mirrors are aligned parallel to each other so that light can be confined between the mirrors, forming a standing-wave resonator for electromagnetic waves. The cavity is considered to be on resonance when the cavity length, $L_{cav}$, is equal to an integer number of half-wavelengths:

(14) $$L_{cav}=m\left(\frac{\lambda_{c}}{2n}\right),$$

where $\lambda_{c}$, n, and m are the wavelength of light, the RI of the material inside the cavity and the mode order, respectively.

The main elements that constitute a Fabry–Pérot cavity are two reflective surfaces, e.g., metal (Ag, Al, or Au) mirrors. The thickness of the adjustable spacing determines the resonance frequency of the cavity. The bottom reflective surface is usually supported by a transparent substrate (e.g., glass or quartz in the case of visible light, and CaF_2_ or ZnSe in the case of infrared light). An alternative to these reflective surfaces is distributed Bragg reflectors (DBRs), which are multilayer structures comprising alternating quarter-wavelength layers with low and high RIs with a thickness of λ/4.

Generally, a high Q-factor of the cavity is achieved by improving the reflectivity of the mirrors and can be calculated using Equation (5). The use of DBRs helps increase the Q-factor of the cavity, which, in this case, depends on the number of layer pairs [77] and the RI contrast between the two dielectric materials. However, they require advanced manufacture methods that are usually time-consuming. At the same time, metal mirror microcavities have a relatively low Q-factor, which is limited by the intrinsic reflectivity (R) of the metals (R_Al_ ~ 90–85%, R_Ag_ ~ 95–99%, and R_Au_ ~ 70–90% in the range of 550–700 nm [78,79]) and the thickness of the films, e.g., gold colloid films, as a function of metal volume fractions [80]. Surface roughness of the metal mirrors can lead to a deterioration of the optical response and a shift in the peak wavelengths of resonance modes compared with smooth mirrors [81]. In addition, when metal mirrors are used, surface plasmon resonance (SPR) can be observed as a result of the contact of the metal mirror with the atoms or molecules inside the cavity. SPR can cause distortion of mode dispersion curves, resulting in an imprecise assessment of the SC impact on the properties of atoms or molecules [82]. Therefore, a thin protection layer is recommended to avoid inaccurate evaluation of strong coupling and protect the mirrors from damage. Moreover, a setup that allows the study of different samples and the adjustment of the cavity length depending on the required wavelength is desirable. For this propose, tunable Fabry–Pérot cavities have been developed that allow for varying the length of the cavity depending on each particular sample, thus making it flexible and more versatile, avoiding the need to design a new setup for each sample [83].

Apart from regular Fabry–Pérot cavities, cavities filled with a liquid medium, which have been considerably less studied, are of much interest. However, as shown in Equation (14), it is technically challenging to fabricate a cavity with the sizes required for ensuring resonance conditions, especially for electronic transitions. The energy of electronic transitions is in the visible range; therefore, a nanosized cavity is required. At the same time, strong coupling to molecular vibrations is more feasible because it requires a micrometer-scale cavity length.

**Figure 7:**
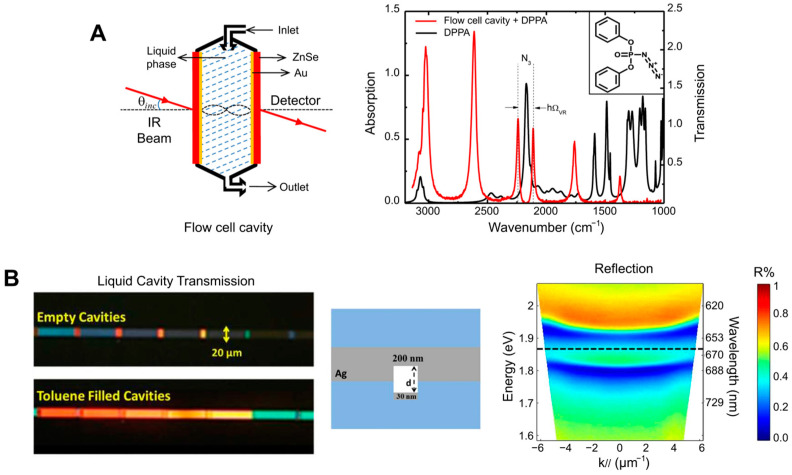
(**A**) (**Left panel**): schematics of the flow-cell Fabry–Pérot cavity. (**Right panel**): infrared absorption spectrum of DPPA (black) and its transmission spectrum under strong coupling with the Fabry–Pérot cavity modes (red). (**B**) (**Left panel**): optical micrographs showing white light transmission through the nanofluidic Fabry–Pérot cavities before and after filling them with toluene. (**Central panel**): schematic cross section of Fabry–Pérot nanocavities of thickness d, made of Ag layers and glass substrates. (**Right panel**): angle-resolved dispersion reflection spectra of a Fabry–Pérot nanocavity containing chlorin e6 in dimethylformamide at a high concentration under a strong coupling regime. Adapted with permission from George, J., et al. (2015) [21], published by the American Chemical Society (**A**), and Bahsoun, H. et al. (2017) [84], published by the American Chemical Society (**B**).

Figure 7 shows different setups used to implement micro-/nanofluidics under the conditions of strong coupling. Generally, such systems consist of a Fabry–Pérot cavity with two microfluidic junctions. The first study on strong coupling in a liquid phase was published in 2015 [21] (Figure 7A). There, a tunable microcavity was used to obtain vibrational strong coupling in the infrared range for a variety of functional groups. The setup consisted of a commercial liquid sample cell unit with ZnSe windows coated with a 10 nm Au layer and separated with Mylar spacers. Different substances were studied, including diphenyl phosphoryl azide (DPPA) with an isolated N=N=N stretching mode at 2169 cm^−1^. The results show a vacuum Rabi splitting of about 127 cm^−1^, which is larger than both the FWHM of the cavity mode (43 cm^−1^) and the vibrational band (39 cm^−1^).

Another example of a microfludic Fabry–Pérot microcavity is presented in [13]. Here, an infrared microfluidic cavity was used to investigate cooperative VSC of the solute (p-nitrophenyl benzoate) and solvent (isopropyl acetate) molecules. The reflective surfaces consisted of BaF_2_ substrates sputtered with Au (10 nm), separated by an elastic Mylar spacer (12 μm). VSC was achieved by coupling the cavity mode to the C=O stretching band (1739 cm^−1^) of isopropyl acetate at a solute concentration 100 times lower than that of the bulk solvent. The cooperative VSC of the transesterification reaction decreased the free energy of activation by 2–5 kJ mol^−1^, thereby catalyzing the reaction.

In [85] VSC was achieved using the ferrocyanide ion [Fe(CN)_6_]^4−^ as a model substance in an aqueous solution. The reflective surfaces of the Fabry–Pérot cavity consisted of two rectangular CaF_2_ glasses coated with Au. Both glasses had sizes of l = 38.5 mm, w = 19.0 mm, and h = 4.0 mm, and one of them had 2 mm through holes drilled to form a microfluidic channel. The results showed tunable strong vibrational coupling of [Fe(CN)_6_]^4−^ at moderate concentrations, with a lower limit of about 15 mM, because of its large molar extinction coefficient and narrow resonance bandwidth. The C≡N stretched at 2038.6 cm^−1^ split into two polariton peaks ~90 cm^−1^ apart in a saturated (0.649 M) K_4_[Fe(CN)_6_] solution, with a cavity length of 6.84 μm.

By functionalizing the sensing area with receptor molecules, the optical cavity can be adapted for biosensing. In [86], a cavity-based optical biosensor was adapted for biosensing experiments with streptavidin and C-reactive protein. The sensor detected the local RI change due to the adsorption of target biomolecules on the receptor molecules. When the target biomolecules were adsorbed on the receptors, a shift in the resonant response occurred. The fluid control was performed using a small sample volume of 15 µL for 30 min. The results showed limits of detection (LODs) of 1.35 nM for streptavidin and 377 pM for C-reactive protein.

Nanofluidic Fabry–Pérot cavities used to study the photophysical properties of the chlorophyll analog chlorin e6 (Ce6) in both the weak and strong coupling regimes are shown in Figure 7B. The reflective surfaces consisted of two parallel Ag layers on 2.5 cm × 2.5 cm glass substrates. The distance between the mirrors was on the order of 100 nm. The nanofluidic channel (a rectangular area of 10–20 µm × 120 µm) was formed using a focused ion beam. Then, a 30 nm Ag layer was evaporated on the substrate to act as the channel bottom mirror. The two substrates were cold-welded by high pressure (~1.6 tons/cm^2^). The thickness of the top mirror layer depended on the required measurements. For instance, a 30 nm top Ag mirror was used in the transmission mode and a 200 nm one in the reflection mode. Here, solutions of chlorin e6 in dimethylformamide (DMF) at a high concentration (0.18 M) were introduced into the nanocavities by capillary forces. The mode splitting (Figure 7B, right panel) was due to the strong light–matter coupling and had a value of about 110 meV [84]. With these findings, the research group demonstrated how to obtain nanoscale liquid Fabry−Pérot nanocavities to study strong light–matter coupling for molecular solutions.

**Figure 8:**
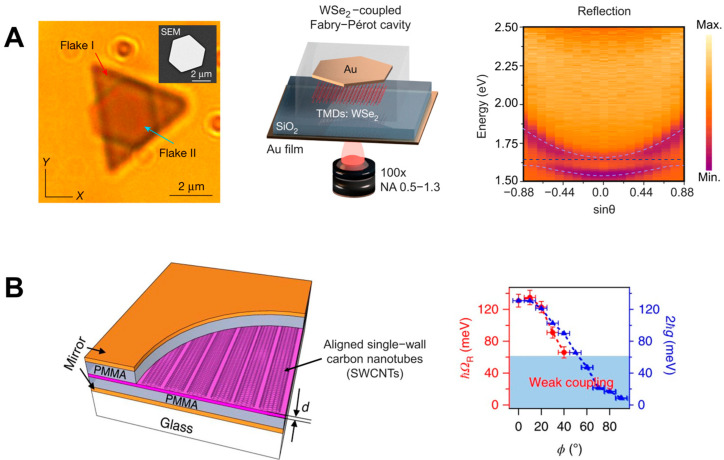
(**A**) (**Left panel**): Two parallel gold nanoflakes floating in an aqueous solution of a ligand (CTAB). Bright-field image of a self-assembled dimer formed from flake I (triangular) and flake II (hexagonal). Inset: a SEM image of an exemplary gold nanoflake. (**Central panel**): a self-assembled microcavity coupled to a few layers of WSe_2_. (**Right panel**): anti-crossing of self-assembled microcavity coupled to a few layers of WSe_2_. (**B**) (**Left panel**): schematics of the exciton–polariton device based on a film (with the nanoscale thickness *d*) of aligned carbon nanotubes. (**Right panel**): the vacuum Rabi splitting $\left(\hbar \Omega_{R}\right)$ (red dots) and the extracted coupling strength (2ℏg) (blue triangles) continuously tuned via the polarization angle ϕ from strong to weak coupling. Adapted with permission from Munkhbat et al. (2021) [24], published by Springer Nature (**A**), and Gao et al. (2018) [94], published by Springer Nature (**B**).

In addition, new avenues in strong light–matter coupling have been explored upon the incorporation of 2D materials [87,88,89,90,91], such as self-hybridized exciton–polaritons obtained using Fabry–Pérot microcavities or plasmonic nanoantennas [92]. Two-dimensional materials are atomically thin materials that exhibit unique properties due to their reduced dimensionality. These materials offer novel electronic, optical, and mechanical properties, which makes them highly attractive for applications in nanophotonics, optoelectronics, and quantum technologies. When these materials interact with light, the confinement of electrons within two dimensions leads to enhanced light–matter interactions. In particular, strong light–matter coupling can occur, which is of great interest for advancing the development of new photodetectors, solar cells, and quantum emitters. Other new configurations for obtaining strong coupling have also been studied. For example, self-assembled optical Fabry–Pérot microcavities called Casimir cavities can have cavity lengths down to 100–200 nm, enabling SC in the visible range in a tunable regime. Figure 8A shows two possible configurations of Casimir cavities, which are either two parallel nanoflakes floating in an aqueous solution or a nanoflake placed above a static mirror. The system loaded with a several-layer WSe_2_ flake under strong coupling conditions exhibited anti-crossing (see Figure 2) with a Rabi splitting of $\Omega_{R}\approx$ 110 meV [24]. In addition, the control of objects with a nanometer precision was performed using the critical Casimir repulsion forces to counteract the friction due to the Casimir–Lifshitz forces preventing the contacting surfaces from moving [93]. These results offer an option to fabricate liquid nanocavities with a nanometer precision where maintaining the parallelism of two flat surfaces at small distances has proven to be difficult.

Light–matter coupling has also been observed on a carbon nanotube in a Fabry–Pérot microcavity based on a uniform nanoscale-thick film of aligned, packed, single-chirality nanotubes, as shown in Figure 8B. The system displays ultrastrong coupling (with VRS as high as 329 meV or a coupling strength to transition energy ratio of 13.3%) for polarization parallel to the nanotube axis [94]. The system is tunable from weak coupling to strong and ultrastrong couplings. However, the main applications of the system are related to ultrastrong coupling, which is beyond the scope of this review. These systems have been reviewed in detail in Refs. [95,96,97].

### 4.2. Photonic-Crystal Fiber Plasmonic Sensor

The design of a photonic-crystal fiber (PCF) plasmonic sensor combines the concepts of PCF and plasmonic materials. A PCF is a type of optical fiber that leverages the properties of photonic crystals, periodic structures characterized by a photonic bandgap preventing waves of specific lengths from propagating in the cladding, thus confining them to the core. This effect is determined by a characteristic microstructure, which consists of an organized pattern of air channels running along the entire fiber, designed to confine and guide light. By incorporating a plasmonic material layer into the PCF structure, surface plasmon resonance is enabled, which improves the sensitivity to environmental changes at the metal–dielectric interface. Typically, this involves the deposition of a metal layer, such as gold or silver, on the inner surface of the air channels or on the outer surface of the fiber, and the dielectric material is the medium surrounding the fiber, such as air or a specific analyte solution.

**Figure 9:**
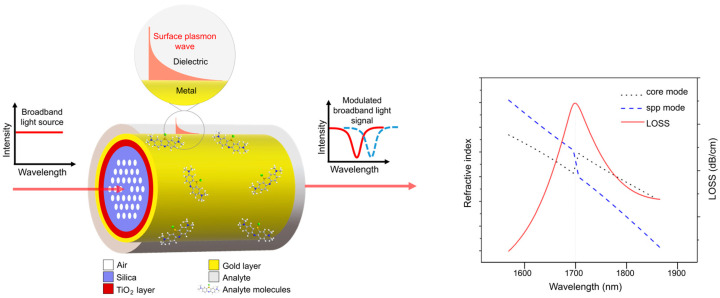
Schematic representation of a photonic-crystal fiber surface plasmon resonance sensor. (**Left panel**): the sensor with an evanescent wave generated by the surface plasmon wave propagating at the metal–dielectric interface. (**Right panel**): the real part of the effective index for the core and surface plasmon polariton (spp) modes and the loss spectrum of the core mode. The wavelength at which complete mode coupling occurs (1700 nm) is indicated.

As shown in Figure 9, the PCF surface plasmon resonance (SPR) sensor employs the evanescent wave generated by the surface plasmonic wave (SPW) propagating at the metal–dielectric interface. This occurs when the frequency of incident light matches the oscillation frequency of surface electrons. The coupling between the SPW and the evanescent field at a specific wavelength creates the resonance condition, leading to the appearance of the confinement loss peak [98]. The confinement loss is calculated from the imaginary part of the $n_{eff}$ (effective refractive index) values of the core-guided mode:

(15) αlossdBcm=8.686×2πλ⋅Im[neff]×104,

where λ is the operating wavelength and Im[neff] is the imaginary part of the $n_{eff}$ of the core-guided fundamental mode, the primary mode of light propagation in PCF. More metrics and parameters are presented in Section B of the Supplementary Materials.

Due to the sensitivity of the SPW to the RI of the surrounding medium, even a small change in the RI caused by an analyte leads to a shift in the resonance peak. Therefore, these sensors are suitable for applications in medical diagnosis, biological and chemical sensing, and environmental monitoring based on RI measurement [99].

Due to their microchannel structure allowing for strong coupling between light and the liquid medium inside, PCFs can be used for both light guiding and fluid transport. The channels in the cladding region of the PCF offer the possibility to impregnate it with biological samples or chemicals in the gaseous or liquid form, which can not only penetrate into the fiber core but also fill the internal and external sensing channels of the fiber. Additionally, the sensitivity and selectivity of the PCF SPR sensor could be improved by functionalizing the microchannels of the PCF with capture molecules binding biomolecular targets.

The main benefits of combining PCF with the SPR technology are the ability to detect a broad spectrum of analytes under a variety of conditions (making them suitable for various applications), compact size (which is important in nanosensors and remote sensing applications), and extremely high sensitivity. Moreover, PCF sensing requires very small volumes of the analyte (from several tenths to several tens of microliters) due to the small size of the air channels of the PCF core and cladding, as well as an intensified interaction between the sample and light. This possibility of using extremely small sample volumes is especially valuable for chemical and biological applications, such as identifying toxic chemicals, antibodies, blood components, cells, bacteria, DNA, and viruses [100]. However, a disadvantage of the PCF technology is its extremely high cost and a challenging fabrication process.

There are various types of PCF plasmonic sensors, each designed for a specific sensing application by modifying the structure of the PCF and the plasmonic element configuration. The material of plasmonic element (silver, gold, aluminum, copper, bismuth, palladium, etc.) also affects the sensitivity of the sensors [101]. For example, compared with other metals, gold exhibits a higher chemical stability and provides a greater analyte-induced change in the position of resonant peak; silver provides a sharper resonance peak, but it is unstable chemically [102]; and copper oxidizes in an aqueous environment [103]. PCF enables both SPR and localized SPR (LSPR) [104,105,106], which differ in that SPR occurs at continuous or periodic metal surfaces, e.g., planar films, gratings, nanohole arrays, etc., whereas LSPR is strongly localized by metal nanostructures: nanospheres, nanodisks, nanorods, nanowires, etc. [107]. The parameters of the nanostructures, such as geometry and material, determine the resonant frequency of LSPR. For instance, aluminum-based nanostructures are used to obtain LSPR in the UV and blue spectral ranges; silver-based structures, in the range from blue to green; and gold is used for LSPR in the range from red to near-infrared. LSPR-based systems have been used for surface sensing, enhanced photocatalysis, and photochemistry [108]. When combined with PCFs, they become particularly suitable for analyzing biological samples and SERS enhancement. The limit of detection (LOD) of LSPR is 0.102 ng/mL for zearalenone in sensors using gold nanoparticles ~25 nm in size [109]. In the case of SPR on a continuous gold film in a sensor chip fabricated on the basis of a self-assembled monolayer, the LOD for zearalenone was 7.07 ng/mL [110]. It has been demonstrated that, with increasing the number of deposited nanoparticles, the changes in the LSPR maximum decrease but the wavelength shifts increase [111]. These results show that LSPR is more sensitive to molecular binding events at the surface due to its localized field enhancement. However, it is still difficult to deposit nanoparticles uniformly along the optical fiber.

Innovative sensor designs have been proposed that use diverse configurations, e.g., photonic quasicrystal fibers (PQFs) characterized by ordered but nonperiodic arrangement of air channels in the cladding region, unique optical waveguide properties, such as birefringence, a low dispersion, and a high confinement loss.

To further improve sensing capabilities, multistructural forms have been used and techniques for coating PCF with thin metal layers (e.g., gold or silver) have been improved to enhance the performance of PCF SPR sensors. In general, a good sensing performance requires a uniform and thin metal coating for reducing the surface roughness, hence ensuring laminar analyte flow. An alternative way to form a plasmonic coating in a PCF sensor is the use of metal nanoparticles synthesized by colloidal methods under the conditions ensuring the control of their size. For example, gold nanoparticles can be synthesized using the citrate reduction method. By controlling the concentration of citrate and the reaction conditions, the size of the gold nanoparticles can be precisely controlled [112]. In addition, the metal layer can also be coated onto the inner surface of the PCF to create a plasmonic layer. Typically, two methods are used to deposit the sensing metal layer in PCFs: chemical vapor deposition (CVD) [113,114] or high-pressure metal pumping [115,116].

**Figure 10:**
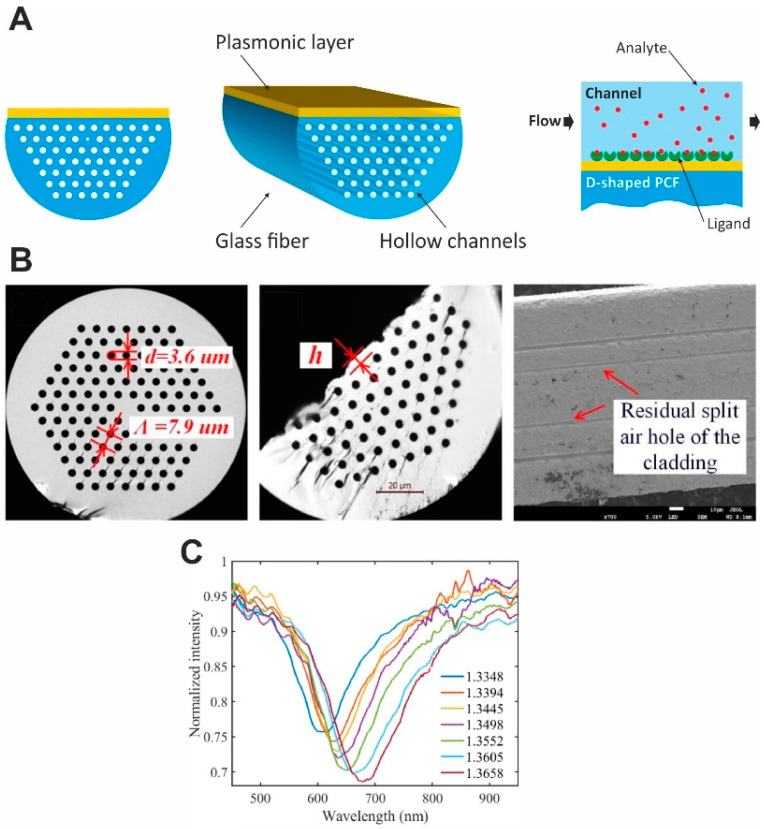
(**A**) Schematic representation of a D-shaped photonic-crystal fiber (PCF) surface plasmon resonance biosensor for refractive index sensing. (**Left panel**): PCF cross section. Central panel: PCF general view. (**Right panel**): schematics of the sensor operation. (**B**) (**Left panel**): a micrograph of the PCF cross section before polishing. Central panel: the cross section of the D-shaped sensor. (**Right panel**): a SEM image of the polished and Au-coated D-shaped PCF. Split air holes can be seen. (**C**) Experimentally detected spectrum at different RIs. Adapted with permission from Luo et al. (2023) [123], published by Elsevier (**B**,**C**).

Because the air holes to be coated with metal are only a few micrometers in size, it is challenging to coat their inner surface with metal while maintaining the homogeneity of the film. To overcome this fabrication difficulty, a D-shaped PCF configuration (Figure 10) has been proposed for an external sensing approach. These sensors are fabricated by flattening one side of the PCF (thus creating a D-shaped cross-section) and coating the flat side with a metal layer. In the case of a D-shaped sensor, the “stack and draw” approach or capillary stacking technique could be used for the fabrication [117]. For the formation of air channels, capillaries are stacked to form the core of the PCF, which is then drawn to obtain a PCF cane, after which a protective tube is added to the surface of the PCF to achieve the desired sensor structure. Afterward, a flat surface is obtained by polishing the upper half of the fiber [118], and a micro-opening channel is formed by focused ion beam milling or femtosecond laser micromachining. In order to form the SPR film in the channel, chemical vapor deposition of metal is used [119]. In addition, atomic layer deposition (ALD) could be considered for applying a gold layer onto curved surfaces. Finally, the bio-analyte is applied onto the metal layer for subsequent measurements [120]. Several D-shaped PCF SPR biosensors, including those based on 2D plasmonic materials, such as graphene and MoS_2_, have been reported recently [89,121,122].

**Figure 11:**
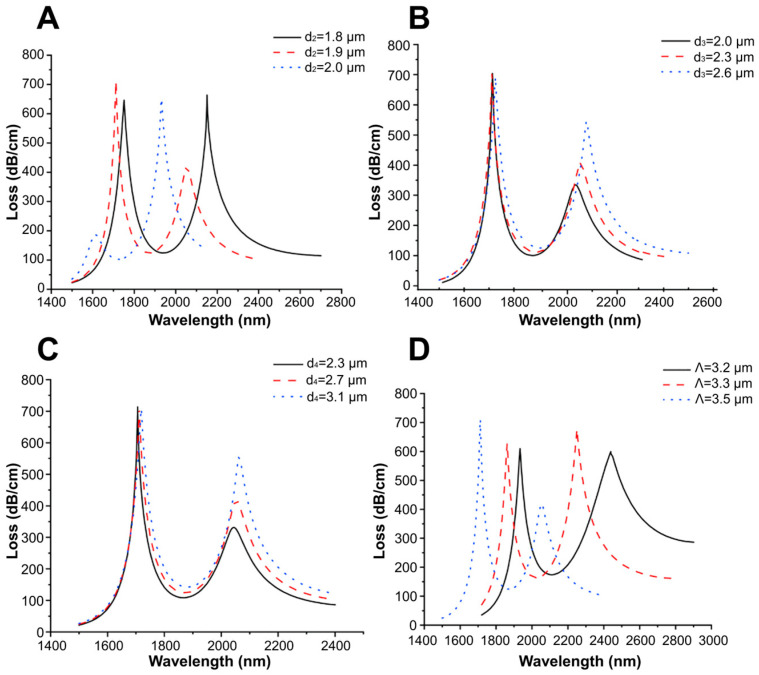
Loss spectra of the sensor with different (**A**–**C**) diameters of the air holes and (**D**) hole-to-hole spacings (Λ). Adapted with permission from Ma et al. (2023) [124], published by Elsevier.

Later, the D-shaped sensor was theoretically adapted for simultaneous detection of changes in the RI in two separate wavelength ranges; the adapted sensor is referred to as a dual-band D-shaped PCF SPR sensor [124]. In this case, a Au-TiO_2_ grating is deposited onto the flat surface of a D-shaped PCF, and then, SPRs are excited for detecting the change in the RI of the analyte by analyzing the loss spectra and birefringence. The maximum sensitivity of the sensor is 26,700 nm/RIU and the RI resolution is 3.75 × 10^−6^ RIU. Furthermore, a Rabi-like loss spectrum splitting (Figure 11) was simulated by making a small hole in the fiber. In this case, Rabi-like splitting results in two hybrid modes (loss peaks) with higher and lower frequencies. It has been shown that the two loss peaks allow for the detection of RI changes in the corresponding spectral range, which makes the sensor even more useful.

The integration of PCF SPR sensors with microfluidic systems has ensured precise control of sample introduction and enhanced sensor functionality. It should be noted that the sample solution filling the microfluidic channels of the PCF should flow in the same direction as the light propagates during sensing. Therefore, misaligning the PCF device can affect the coupling condition and distort the results. This issue can be addressed by using an external microfluidic channel, which, in addition, simplifies the fabrication. For example, an external microfluidic module can be added to a D-shaped PCF sensor to obtain a sealed system where the solution on the surface of the metal coating can be replaced without disturbing the internal setup. Moreover, functionalization of PCF microchannels with biomolecules improves the selectivity and sensitivity of PCF SPR sensors for biological applications. The fabrication of PCF SPR sensors is described in detail in Refs. [89,92]. Recently, PCF-based biosensors with single-channel, semi-channel, and multi-channel configurations have been proposed for detecting analytes with different RI values [125,126,127]. In addition, the application of machine learning algorithms to the analysis of PCF SPR sensor signals is used to improve the accuracy and reliability of measurements.

Theoretical and computational research is being carried out in order to develop highly sensitive and portable PCF SPR sensors. For example, the finite element method (FEM) is used to determine the optimal geometric parameters of such systems [128,129]. In [130], two different configurations of RI biosensors based on photonic quasicrystal fibers (PQF-RIBS) with six- and eightfold air hole patterns have been simulated by the FEM. The results show that, for the sixfold configuration, the maximum sensitivity is 28,000 nm/RIU for an analyte RI range of 1.41–1.42, the figure of merit being 491. In the case of the eightfold configuration, the maximum sensitivity is 27,000 nm/RIU for an analyte RI range of 1.40–1.41, the figure of merit being 231. Both systems operate in a wide range of wavelengths, from 600 to 1300 nm. In the system with an eightfold arrangement of the air holes, the *n_eff_* is reduced and confinement loss is increased compared with the system with a sixfold one. This also indicates a strong mode–field coupling in the eightfold PQF-based RIBS.

Thus, the combination of biosensors based on PCF or PQF with microfluidics has already been tested experimentally, with theoretical and experimental research still underway in order to further improve the sensing properties of these devices.

### 4.3. Plasmonic Nanocavity

Surface plasmons are collective electron oscillations that arise at the interface between two materials, e.g., metal and dielectric, in such a way that the real part of the dielectric function changes its sign across the interface [131]. Plasmonic nanocavities are optical structures that use surface plasmon resonance to confine light at the plasmonic resonance frequency. Plasmon resonance could be either localized (e.g., in metal nanoparticles) or delocalized (e.g., in thin metal films) [132]. These structures are used to obtain strong enhancement of local electromagnetic fields, required for coupling with target molecules. The EM field is enhanced at certain locations, such as tips, sharp corners, or junctions in plasmonic nanoparticles (NPs), generating focused “hot spots” with sizes similar to those of single molecules. Therefore, although plasmonic nanocavities have low Q-factors and high ohmic losses, these structures are particularly attractive for achieving strong field confinement with a subwavelength effective mode volume (Figure 4), making it possible to obtain strong coupling at the level of one or several molecules [133]. The physical sizes of plasmonic cavities can be smaller than the diffraction limit of light, in contrast to those of conventional cavities, which are limited by the wavelength. However, it is important to note that the coupling strength is determined mainly by the dipole moment of the analyte and its concentration in the mode volume, regardless of the cavity type [38].

Theoretically, plasmonic nanocavities are usually described in terms of the Jaynes–Cummings model considered in the previous section. The coupling strength of a system (e.g., a dipole) to an isolated nanocavity can be expressed as

(16) $$2g=\sqrt{(\Gamma_{tot}-\Gamma_{0})\kappa },$$

where $\Gamma_{tot}$ is the decay rate of the system coupled to the isolated nanocavity, $\Gamma_{0}$ is the decay rate of the system in free space, which is determined as the sum of its radiative and nonradiative decay rates, and *κ* is the total loss rate of the cavity $\left(\kappa =\kappa_{rad}+\kappa_{nrad}\right)$ (see Section C of the Supplementary Materials).

Experimentally, since the demonstration of the confinement of light in nanometer gaps between metal nanoparticles, plasmonic nanocavities have been employed for enhancing the Raman scattering signal [134,135] and the photoluminescence or fluorescence intensity [136,137], thus substantially increasing the sensitivity of plasmonic nanocavities. Later, their performance has been improved by integration with conventional photonics [138]. With the demonstration of strong coupling between light and matter in plasmonic nanocavities and the improvement of fabrication techniques, such as electron beam lithography and focused ion beam milling, more precise and complex plasmonic nanocavity designs became feasible, including the integration with microfluidics. In order to confine light efficiently, a plasmonic nanocavity should match a number of parameters, such as the optimal roughness of metal structures [81], energy loss, reactivity, and resonant region (plasmon tuning range) [131]. There are various configurations combining plasmonic nanocavities with microfluidics, including isolated plasmon cavities, dimers, arrays of nanoparticles called plasmonic lattices, and nanoparticle-on-mirror configurations [139,140], which are described below.

**Figure 12:**
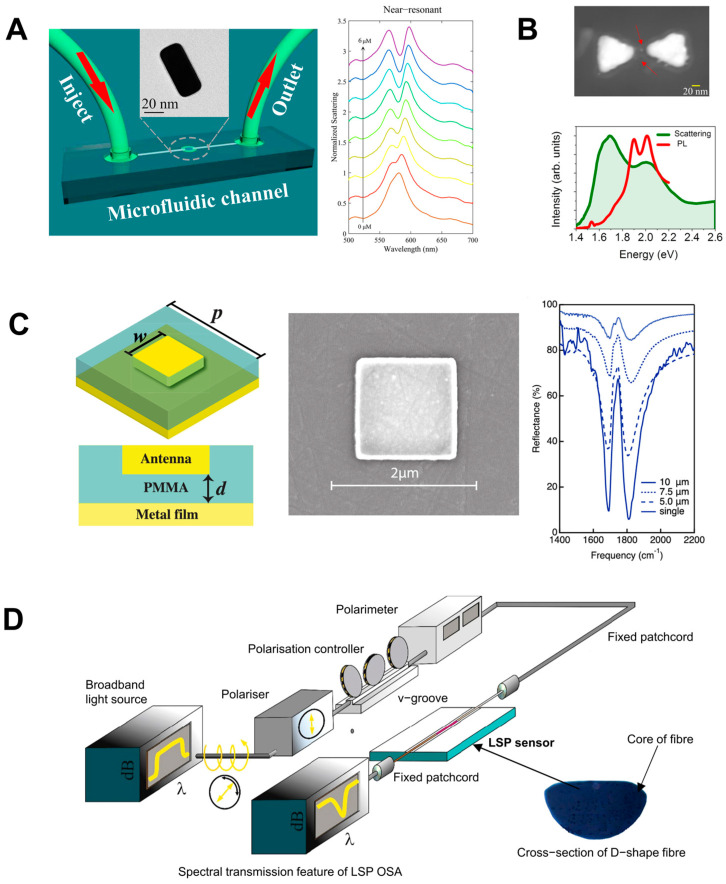
(**A**) (**Left panel**): schematics of the nanostructured microfluidic device for detection of H_2_O_2_. (**Right panel**): dynamic tuning of the plasmon−exciton coupling in the microfluidic channel: emergence and evolution of hybrid polariton states in scattering spectra. Colors show the concentrations of J-aggregates adjusted from 0 to 6.0 μM (from the bottom up, the concentrations are 0, 0.5, 1.0, 1.5, 2.0, 3.0, 4.0, 5.0, and 6.0 μM) (**B**) (**Top panel**): STEM images of a device with two quantum dots trapped within plasmonic bowties. The red arrows indicate the QDs in the bowtie gaps. (**Bottom panel**): the dark-field scattering (green) and photoluminescence (PL) (red) spectra of the device. (**C**) Left panel: schematics of the nanogap patch antenna. (**Central panel**): a SEM image of the fabricated patch antenna (top view). (**Right panel**): measured reflectance spectra of the nanogap patch antenna cavity for different periodicities of the array. The solid curve shows the measured reflectance from a single nanogap patch antenna. (**D**) Schematics of a plasmonic biosensor capable of detecting bisphenol A at ultralow concentrations. Adapted with permission from Liang et al. (2021) [51], published by American Chemical Society (**A**); Gupta et al. (2021) [142], published by Springer Nature (**B**); Dayal et al. (2021) [145], published by American Chemical Society (**C**), and Allsop et al. (2019) [138], published by Elsevier (**D**).

In [51], an ultrasensitive biomechanical nanosensor was proposed, and the coupling of J-aggregates to an isolated cuboid Au@Ag nanocavity in a microfluidic environment was analyzed (Figure 12A). The dye concentration was continuously varied in the nanoscale space adjacent to the plasmon hot spots of the Au@Ag nanocavity to observe the emergence and evolution of light–matter coupling. This tunability supported by microfluidics allowed for obtaining a coupled J-aggregate exciton number from 0 to ~3 and Rabi splitting of up to 167 meV.

The fabrication of dimers by placing gold or silver nanoparticles close to one another has been reported. These nanoparticles can interact with one another through their plasmonic fields, which leads to unique optical properties that differ from those of individual nanoparticles [141]. In [142] QDs were embedded in plasmonic cavities formed by silver bowties on 18 nm SiO_2_ membranes. The QDs are positioned into the gap region of the bowties using interfacial capillary forces. The authors succeeded in increasing the coupling strength by improving the fabrication of the QD–plasmonic cavity device by reducing the thickness of the adhesion layer under the plasmonic cavity. The results have shown coupling strength values in the range of 50–110 meV (Figure 12B). The possibility of further enhancing the coupling to individual QDs could make it possible to control the chemical reactivity, manipulate the excited states, and engineer single-photon sources operating at room temperature for quantum informatic applications.

Moreover, for better tunability and performance, plasmonic nanocavities can be fabricated in the nanoparticle-on-mirror configuration [143]. For instance, nanoantennas have been used to obtain vibrational strong coupling. These systems also could be integrated with nanofluidics [144]. Figure 12C shows the strong coupling of gap surface plasmons and molecular vibrations in an ultracompact hybrid system based on a nanogap patch antenna, with a Rabi splitting as high as 108 cm^−1^. Other studies support the possibility of integrating these configurations with nanofluidics in order to obtain strong coupling of molecules in the liquid phase [145].

Multifunctional plasmonic nanocavities capable of multiple sensing have been engineered by fabricating plasmon ensembles to improve optical trapping and enhancement of the electric field intensity in the light–matter interaction. For example, a plasmonic biosensor for the detection of bisphenol A at ultralow concentrations consisting of an array of gold nanoantennas has been fabricated on the basis of an optical fiber with a D-shaped cladding [138] (Figure 12D). The fluidic cell used a programmable syringe driver equipped with two isolation valves, on the delivery and exit ports of the fluidic cell, to hold and immerse the sensor. The maximum volume of solution contained in the cell was 1.9 mL. The device could detect a small amount of bisphenol A, providing a wavelength shift of 0.15 ± 0.01 nm in response to the analyte concentration of 1 fM, with a detection limit of 330 ± 70 aM.

Moreover, hybrid structures consisting of plasmonic and 2D materials have been developed [87,88,89,91], e.g., an Ag nanoantenna combined with a layered WS_2_ flake [146] and ultracompact plasmonic nanocavities containing WSe_2_ with a Rabi splitting exceeding 135 meV [147]. Another example is a fluidic system consisting of self-organized gold nanocavities enhanced with graphene nanosheets for plasmon-assisted electrochemical detection of H_2_O_2_ released from cancer cells, with an LOD of 1 pM in a linear range of 1 pM–10 μM. Here, Au nanoparticles generated strong electromagnetic fields resulting in a high concentration of electric charge carriers at the electrode surface, and graphene further enhanced the electrode properties [148]. Plasmonic nanocavities strongly coupled to excitons in 2D materials consisting of one or several layers are characterized by room-temperature functioning, miniature size, and high stability.

Strong light–matter coupling has been explored with the use of tip cavities where a plasmonic tip is employed to induce localized light–matter interactions. The nanoscale confinement of light in these configurations leads to extremely small mode volumes, allowing researchers to investigate cavity quantum electrodynamics (CQED) effects. This approach enables precise control of light–matter interactions at the quantum level. Unlike static cavities, the 3D movable tip cavity offers the flexibility to perform control experiments across various emitters in a strong coupling regime. This adaptability introduces a new strategy for developing next-generation nanophotonic devices, overcoming performance limitations of the current optoelectronic technologies [149].

Research in plasmonic nanocavities and microfluidics continues to bring novel applications. However, challenges still remain preventing the use of the full potential of plasmonic cavities, the key ones being the requirements of precisely and controllably positioning molecules in nanosized plasmonic mode volumes, determining the best plasmonic cavity geometry, finding how to separate useful photons from background ones, dealing with the fluorescence quenching in the vicinity of metal particles [48], and precisely determining the plasmon field intensity distribution in the nanogap. In order to prevent molecular aggregation of the emitters and to ensure that the transition dipole moment is perfectly aligned with the gap plasmon (along the electric field), a host–guest chemical approach has been used. An example is the confinement of methylene blue using the macrocyclic cucurbit[*n*]uril molecules. Cucurbit[7]uril is water-soluble and can host only one methylene blue molecule inside. Without the host, the methylene blue molecules oriented perpendicular to the plasmon field exhibit only a gap plasmon [150]. In contrast, the host enables the alignment of the molecule parallel to the plasmon field, in which case the spectrum exhibits peak splitting due to the strong interaction between the molecule and the plasmon. In addition, residual molecules, such as dyes, in the microfluidic channel may bring ambiguity in analyzing the coupling process. Therefore, to minimize the influence of uncoupled dye, the microfluidic device is flushed several times with deionized water before spectral measurements. Simulation of the electric field predicts that only the dye molecules attached closely to the nanotips could contribute to the strong coupling process, while the other molecules should have a low impact on the hybrid system [51]. In addition, strong coupling has been theoretically predicted between semiconducting single-walled carbon nanotubes and metal nanoelectrodes, with a Rabi splitting of ℏΩ≈ 127 meV and an augmented Purcell effect (*F* = 105) at a source-drain voltage voltage of 1.75 V. These results show that a strong coupling regime and boosted Purcell effect can be reached in electrically driven plasmonic junctions [151], which could be another option for combining with fluidics.

### 4.4. Whispering-Gallery Mode Microcavity

**Figure 13:**
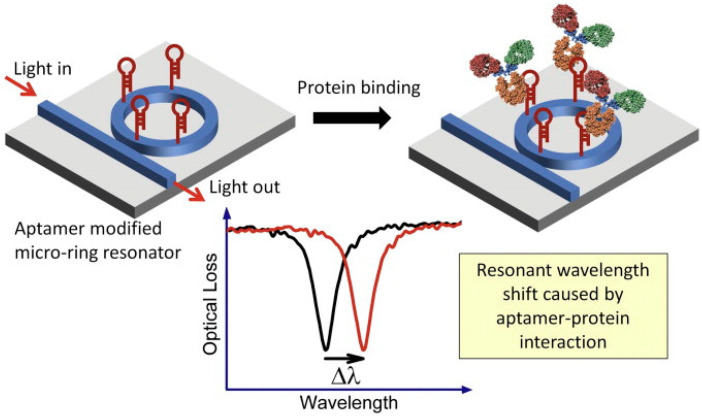
A schematic diagram of the principle of a whispering-gallery mode microcavity (microring resonator) used for biosensing and the corresponding optical loss spectrum. Reproduced with permission from Park, M.K. (2013) [152], published by Elsevier.

In a WGM microcavity, light propagates in the form of circulating waveguide modes confined in a microscale volume through total internal reflection and constructive interference on a closed concave surface. This light is trapped into the cavity via an adjacent linear waveguide positioned within a couple of hundred nanometers. Part of the EM field confined on the closed concave surface extends beyond the cavity surface; this part is referred to as the evanescent field. Regarding photonic crystal fiber plasmonic sensors, optical WGMs are highly sensitive to the surrounding medium (analyte solution), and the changes around the evanescent field can lead to changes in the resonance mode. Therefore, when an analyte interacts with the evanescent field, it alters the local RI. For example, biomolecule binding (e.g., antibody–antigen interaction) on the surface of the cavity leads to a change in the effective RI and a shift of the resonant wavelength (Figure 13). This effect is used for biochemical, temperature, and mechanical sensing.

Due to the strong light confinement, WGM microcavities have been demonstrated to be excellent candidate devices for obtaining strong coupling between an optical mode and a system [153,154]. These cavities are characterized by their high Q-factor (up to 10^11^), extremely small mode volume, and high optical energy density and have been used for detection and highly sensitive measurements on the microscale and nanoscale, including single-molecule detection.

The main parameters analyzed in WGM microcavities, in addition to the Q-factor (Equation (5)) and mode volume (Equation (7)), are the optical resonance condition and sensitivity. The optical resonance condition for WGMs is as follows:

(17) $$m\lambda =2\pi R_{b}n_{eff},$$

where m is an integer number, λ is the wavelength, $R_{b}$ is the radius of the resonator, and $n_{eff}$ is the effective RI. WGM microcavities are characterized by a high sensitivity, which is important for numerous applications. When a molecule lands on the surface of the microcavity, the circulating light interacts with it, generating the detection signal with a time resolution of microseconds suitable for label-free detection. WGM microlasers exhibit superior label-free detection capabilities, which means that they can detect biological and chemical interactions without the need for external tags or fluorescent markers. This is particularly valuable in real-time monitoring of live biological systems where markers could interfere with natural processes. The integration of gain media (e.g., quantum dots, dyes, or rare-earth ions) into WGM systems transforms them into WGM microlasers. These active systems, unlike passive resonators, can amplify the optical signal, making it easier to detect very small changes, such as molecular binding events. This improves the overall sensitivity and precision of the biosensors, potentially enabling single-molecule detection, which is a substantial breakthrough in various fields, such as diagnostics and environmental monitoring [155].

**Figure 14:**
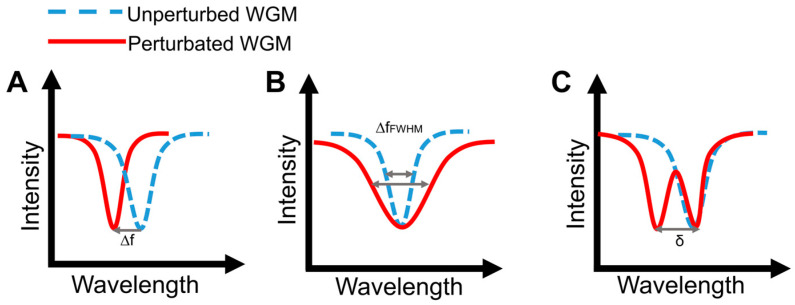
Regimes of sensing using whispering-gallery mode (WGM) microcavities: (**A**) mode shifting, (**B**) mode broadening, and (**C**) mode splitting.

WGM microcavities are fabricated with various geometries, sizes, and materials [156,157]. The most common WGM structures for sensing are microbottles [158,159], microdisks [160,161], microspheres [162,163], microtoroids [164], microcapillaries [165,166], and microrings [167,168]. Table 1 summarizes the main methods used for the fabrication of different WGMs. The sensing in these cavities could be based on mode shifting [169], broadening [170], or splitting [154], as shown in Figure 14.

Mode shifting (Figure 14A) occurs when the effective RI is altered with changes in the surrounding medium. For example, a red shift usually results from an increase in the concentration. The changes in the effective RI can also be influenced by the variations of the temperature and magnetic field due to the thermo-optical effect and magneto-optical effect, respectively. Variation of the geometry of the resonators can be caused by changes in the pressure, flow rate, stress, tension, and thermal expansion [174]. For example, in the case of microbubbles, the wall thickness is selected depending on the practical application: a thin wall is always used for the detection of media inside the microbubble, whereas a thick wall is used for physical sensing, such as temperature sensing. In addition, to avoid errors when different substances in a solution cause the same change in the bulk RI, receptor molecules are integrated for selective detection. This method is sufficiently sensitive for monitoring single-molecule biochemical reactions [175]. Because the sensor sensitivity can be affected by external perturbations, packing is used to enhance stability. For example, glass scaffolds and low-RI polymers are used for protecting coupling systems and optical coupling regions, respectively. The process involves a portable, low-cost fiber fusion splicer for electrode-discharge heating [176].

In mode broadening sensing (Figure 14B), the detection is performed by monitoring the WGM linewidth [170]. The spectral linewidth of the resonance mode is related to the Q-factor, as seen in Equation (5), and changes in the Q-factor are caused by variations of the optical loss. Therefore, the spectral linewidth increases with increasing cavity losses, such as scattering and radiation losses. In contrast to the wavelength shift, mode broadening sensing is unaffected by changes in the external medium due to its self-referenced mechanism. Therefore, it has smaller detection limits for small molecules and single nanoparticles.

Mode splitting (Figure 14C) occurs in WGM microcavities due to the propagation of degenerate modes clockwise (light circulating clockwise around the cavity) and counterclockwise (light circulating counterclockwise around the cavity) as a result of the scattering of light from subwavelength scatterers, such as nanoparticles, entering the resonator mode volume. As a consequence, the eigenstates are transformed into two orthogonal standing waves in the cavity with a mode splitting in the transmission spectrum [154]. The size and number of the scatterers can be determined by measuring the frequency splitting. In contrast to mode broadening, mode splitting requires a cavity with an ultrahigh Q-factor so that the two modes could be distinguished.

**Figure 15:**
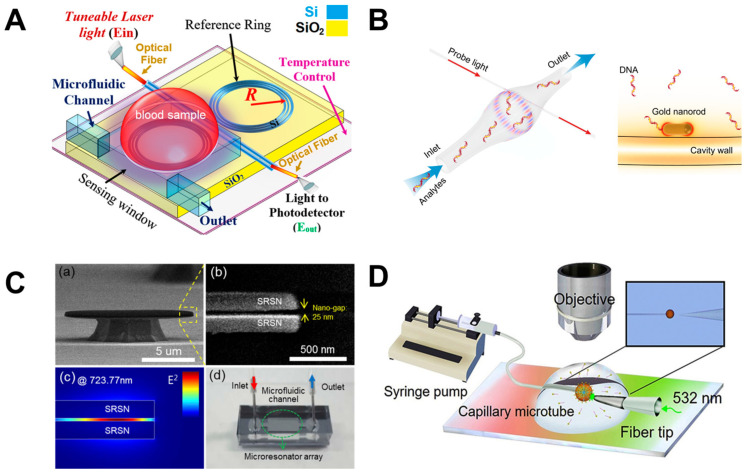
(**A**) Schematics of a ring resonator. (**B**) Schematics of an optofluidic microbubble biosensor. (**Left panel**) Analytes enter the microfluidic channel and are captured by the interior surface of the cavity. (**Right panel**) Schematics of a gold nanorod adsorbed on the interior surface of the microbubble cavity of the Au-coated microsensor. (**C**) (**a**) A SEM image of a silicon disk resonator; (**b**) a magnified image of the silicon disk, with a 25 nm gap indicated; (**c**) simulation of the transverse magnetic fundamental mode in an aqueous environment; (**d**) a photograph of the on-chip sensor integrated with a microfluidic channel; (**D**) schematics of a microdroplet sensor. Reproduced with permission from Bahadoran et al. (2022) [168], published by Springer Nature (**A**); Yu et al. (2022) [181], published by the National Academy of Sciences of USA (**B**); Kim and Lee (2019) [184], published by Optica, formerly known as the Optical Society of America (OSA) (**C**); and Duan et al. (2020) [185], published by Elsevier (**D**).

Generally, WGM devices based on a silicon photonic platform have been developed for multiplexed biological analysis [177]. For example, Figure 15A shows a double-slot-waveguide-based microring resonator for detecting hemoglobin and measuring its concentration in anemia. A sensitivity as high as 1024 nm/RIU with the minimum deflection limit of 14.88 × 10^−6^ RIU was obtained [168].

The versatility of WGMs allows for the integration of additional components, such as 2D materials [89] and plasmonic nanoparticles, ranging from single particles to arrays to obtain optoplasmonic WGM sensors capable of single-molecule detection [178,179]. As mentioned above, localized surface plasmon resonance allows for light confinement beyond the diffraction limit [180], boosting the near-field intensity by a factor of over 1000. Figure 15B [181] shows an ultrasensitive optofluidic biosensor representing a microbubble resonator. The resonator enables the sensing of biomolecules with a detection limit of 0.3 pg/cm^2^ and facilitates single-molecule detection by using plasmonic nanoparticles. This sensor exhibits ultrasmall sample consumption (down to 10 pL) and provides an automated platform for biomedical analysis.

The integration of WGM microcavities with microfluidics enhances their functionality and opens new research avenues in strong light–matter interactions. For instance, silicon ring resonators for high-throughput sensing of specific interactions of biomolecules have been reported [177,182,183]. At the same time, continuous advancements in photonic integration led to the development of on-chip WGM systems, paving the way for compact and efficient photonic devices for communication, sensing, and computation. Figure 15C shows an example of on-chip WGM sensors integrated with microfluidic channels. In this study, silicon nanoclusters were used as microresonators. The silicon disk has a nanogap structure 25 nm in width where the target biomolecules are selectively detected with the sensitivity enhanced by the strongly confined field. The sensitivity confirmed by real-time measurements for the streptavidin–biotin complex is 0.012 nm/nM, which is over 20 times larger than the previously reported WGM sensors with remote readout [184].

Additionally, Figure 15D shows a WGM microdroplet sensor for real-time and high-sensitive detection of acetylcholinesterase and its inhibitors, as well as the acetylcholinesterase enzymatic reaction. For example, this device has been demonstrated to detect pesticides. This device provides the limit of detection as low as 0.1 pg/mL for fenobucarb and 1 pg/mL for dimethoate, which is considerably lower than the standard levels of pesticides specified in water quality standards [185].

Some of the advanced technologies have already been transferred to the industry and are commercially available, e.g., EPIC, ResoSens, and Creoptix WAVE for label-free tests, as well as Maverick. The latter, in particular, is based on silicon photonics, with a single unit including 16 microring resonators, so that 16 analytes can be detected in a single test with a low sample consumption (5–10 µL) within 10 min [186]. The results obtained with the test are in good agreement with standard methods. In addition, the system has a particular advantage over standard clinical tests: it allows to simultaneously perform 12 autoantibody detection assays in whole blood for clinically diagnosed patients in less than 15 min. However, essential bulky elements like the external light source, fluidic pump, spectrometer, and data processing units remain unavoidable [183,187].

Ongoing research aims to achieve and use strong coupling regimes in WGM microcavities for exploring new physical phenomena and improving device performance. Efforts are also made to optimize protocols for the detection of biomolecules. Key challenges include liquid evaporation [29], stability, high sample preparation requirements, clogging of microfluidic channels during the experiment, and ensuring precise geometry of the resonators. A protocol has been developed that encompasses several steps addressing these challenges in the functionalization and detection of protein biomarkers using silicon microring resonator chips, specifically for detecting Ebola virus (EBOV) and Sudan virus (SUDV) soluble glycoprotein (sGP). This protocol can be adapted for other analytes, including cytokines, chemokines, nucleic acids, and some viruses [188].

Different computational tools are used in designing and studying WGMs [189], using FDTD [190], FEM [191], and the Monte Carlo approach [192]. In addition, machine learning has been implemented to automatically extract the spectrum characteristics, with the average accuracies of a single and double parameter identification being 99.5 and 97.0%, respectively [193].

In conclusion, WGM devices offer high sensitivity for biological analysis and versatility, allowing for adding other components, such as plasmonic nanoparticles, that enhance their detection capacity. WGM sensors combined with microfluidics are efficient biomedical devices, used, e.g., in the EPIC and Maverick technologies. Despite challenges such as liquid evaporation and channel clogging, ongoing research and development of advanced computational tools are expected to improve these sensors for broader applications.

## 5. Potential Applications of Strong Light–Matter Coupling in Micro- and Nanofluidics

The light–matter interaction, in particular, strong light–matter coupling, can be employed in various fluidic applications, including monitoring of chemical reactions, modification of chemical pathways, sensing, and nanophotonics. In this section, we discuss the state of the art in some of these application niches.

Control of chemical reactions using new methods, such as polaritonic chemistry and molecular polaritonics, which fundamentally differ from traditional ones, such as electrochemistry, thermochemistry, and photochemistry, attracts much attention from both experimental and theoretical viewpoints. Strong light–matter coupling can modify chemical reactions by altering the energy landscape and the reaction rates. This could be used to improve the efficiency of catalysis, photochemical reactions, and other processes in micro-/nanofluidic systems. However, the multiscale nature of polaritonic chemistry poses unique challenges in both experimental and theoretical research. The discovery of new reaction pathways enhanced by light–matter interactions requires comprehensive experimental study, as well as new theoretical insight and advanced computational models. Dealing with large ensembles of molecules and a large number of degrees of freedom in the optical cavity at wavelength scale requires careful approximations. In addition, even in the case of a single molecule or a small number of molecules under study, the theoretical interpretation of polaritonic effects is challenging because of large loss rates and the atomistic details, i.e., the intricate and specific characteristics of individual atoms and molecules in the system, including their positions, electronic states, interactions, and dynamics. Furthermore, the consideration of quantum effects, such as tunneling, at the nanoparticle–molecule interface requires a multiscale layered technique. This helps to model complex systems where different scales (e.g., atomic, molecular, and macroscopic) are considered simultaneously. This technique allows for the integration of detailed atomic-level interactions with larger-scale phenomena, providing a comprehensive understanding of the system’s behavior.

Advances in machine learning (ML) models trained on ab initio calculations have significantly promoted the study of strong light–matter coupling by improving predictive accuracy and computational efficiency. These ML models, especially deep learning networks, can capture complex patterns in ab initio datasets, enabling precise predictions of the properties of systems experiencing strong coupling. They expedite simulations that would otherwise be computationally intensive, making the study of large and complex systems feasible [194,195]. Recently, a theoretical model based on a combination of a machine learning model trained on ab initio calculations and modular cavity molecular dynamics has been proposed to study the effect of strong light–matter coupling on the Si–C bond in an experimentally investigated S_N_2 reaction. It has revealed a frequency dependence of the rate constant and identified three distinct regimes of kinetic changes [26]. They include a strong inhibition effect without a clear vibrational contribution, vibrationally supported catalysis, and vibrationally supported inhibition, each affecting enthalpy and entropy differently. The findings are consistent with some experimental observations but highlight discrepancies with previous ab initio calculations, suggesting that the dynamic change in electronic polarization induced by nuclear motion and mediated by the cavity is important to consider in the theoretical model.

Chemistry-related applications of strong coupling typically employ Fabry–Pérot cavities and plasmon nanocavities. Photochemical reactions, such as photobleaching, have been demonstrated to be controlled and suppressed by the degree of plasmon–exciton coupling and detuning between organic molecules and plasmonic nanocavities [196]. Detuning refers to the difference in energy between the resonant frequency of the cavity (or plasmonic nanocavity) and the electronic transition frequency of the molecules. By adjusting this energy difference, the strength of interaction between light and matter can be modified to control the reaction dynamics. When the system is precisely tuned, it results in strong coupling and the formation of new polaritonic states that can either enhance or suppress chemical reactions.

Ebbesen’s research group has made pioneering contributions to the field of strong light–matter coupling, elucidating its impact on chemical reactions and molecular properties. In 2012, Ebbesen’s group demonstrated that the spiropyran–merocyanine photoisomerization could be suppressed through strong coupling to a specific electronic transition of merocyanine, resulting in new polaritonic states that alter the energy landscape of the reaction [197]. This discovery highlighted the potential of strong coupling to control reaction dynamics. The group explored vibrational strong coupling (VSC) in ground-state chemical reactions to show that modes of vibrational coupling of molecules to a cavity could enable control of the reaction rates, as demonstrated with the silane deprotection reaction of 1-phenyl-2-trimethylsilylacetylene (PTA) with tetra-n-butylammonium fluoride (TBAF) [22,198]. Additionally, they developed Fabry–Pérot microfluidic flow cells to study reactions in solution under strong coupling conditions [21]. These and other insights pave the way for applications in catalysis and materials science, offering the potential for more efficient and selective chemical processes through fine-tuning of molecular transitions [35,44,199]. Further studies of the effect of VSC on a series of carbonyl reactants undergoing Prins cyclization have shown a significant decrease in the reaction rate [200]. This exploration sheds light on the potential of strong coupling to manipulate and control chemical reactions, including those involving complex ring-forming processes, such as Prins cyclization. Recently, resonant suppression of the intracavity alcoholysis reaction between phenyl isocyanate and cyclohexanol has been reported [73]. Experiments have also shown that, under USC, the interaction between molecular vibrations and photons is intensified, leading to enhanced self-interaction effects within the vibrational modes, which has been found through analyzing the increase in the effective oscillator strength of the asymmetric stretching band of CS_2_. This research provides valuable insights into the intricate dynamics of light–matter interactions at the molecular level and contributes to the understanding of USC effects on molecular vibrations [201]. Another promising direction for advancing catalytic processes is the study of cooperative VSC [45].

Numerous materials have been studied in the strong coupling regime, including J-aggregates [202], organic dyes [203], organic crystals [204], organic semiconducting polymers [205], carbon nanotubes (CNTs) [206], proteins [207], chlorosomes [208], and 2D materials [91,209]. J-aggregates are preferred for their strong interaction with light and cost-effectiveness. Recent research has demonstrated enhanced coupling with MB J-aggregates in plasmon nanocavities, although excessive concentration or increased nanoparticle size reduced the coupling strength [210]. Other J-aggregates, such as the JC-1 cyanine dye, have also been studied [211,212]. Some organic dyes (e.g., BODIPY dyes) have high absorption and quantum yield, but they tend to crystallize, necessitating the use of polymer matrices to prevent inhomogeneity [213]. Organic single crystals in microcavities are preferable for polariton Bose–Einstein condensation due to their structural order and high exciton interaction [214,215]. Organic semiconductor-based microcavities offer high oscillator strengths and flexibility, which makes it possible to fabricate flexible photonic devices that operate at room temperature. However, they face challenges such as environmental degradation, low charge mobility, fabrication difficulties, and thermal instability [205]. CNTs are promising due to their strong exciton–photon interactions and the possibility of functionalization, which extends the potential optoelectronic applications. Specifically, single-walled CNTs (SWCNTs) exhibit narrow-band exciton absorption and strong exciton–photon interactions, making them ideal candidates for generating exciton–polaritons at room temperature [216,217]. Some organic materials, e.g., enhanced green fluorescent proteins, exhibit USC, offering superior photonic properties, such as high quantum yield and photostability. They enable the formation of stable excitons and polaritons at room temperature, surpassing the limitations of traditional semiconductor systems [207]. Research in chlorosomes from photosynthetic bacteria suggests potential applications in artificial light-harvesting devices and the development of “living polariton” systems [208].

Additionally, strong light–matter coupling enhances the sensitivity of micro- and nanofluidic systems by monitoring changes in the optical properties of the system, such as fluorescence, absorbance, and changes in the RI. This enhancement allows for the detection and quantification of analytes with high precision. Nanofluidic systems offer a unique platform for studying strong light–matter coupling due to their capacity for confining and manipulating a fluid flow at the nanoscale. By integrating nanofluidic channels with optical resonators or cavities, a highly efficient real-time sensing platform can be engineered. One of the key advantages of strong light–matter coupling in micro-/nanofluidics for sensing applications is its capacity for enhancing the interaction between light and analytes, leading to better sensitivity and lower detection limits. By tuning the optical properties of the system, researchers can achieve increased signal-to-noise ratio and improved detection capabilities, making strong light–matter coupling a powerful tool for sensing applications with potential in various fields, including healthcare, in particular, point-of-care diagnosis [176], as well as biomolecule sensing, environmental monitoring, and security, where the detection of low concentrations of analytes is critical. However, further research is needed to fully harness the potential of this technology for practical sensing applications.

Sensor systems employing the strong coupling effects can be based on different configurations, including Fabry–Pérot microcavities [86,218] and plasmonic nanocavities [219]. A novel approach to biomolecular detection employs a combination of Fabry–Pérot cavities and surface plasmon photodiodes. This method makes it possible to enhance the sensitivity and specificity of biosensing by leveraging the strong coupling between surface plasmons and the Fabry–Pérot cavity modes. It has been demonstrated that the device can detect low concentrations of antigens, such as the SARS-CoV-2 nucleocapsid protein. This technology holds promise for advancing biosensing applications in medical diagnosis and environmental monitoring by offering a compact, highly sensitive, and versatile platform for detecting a wide range of biomolecules [220].

Recently, a cavity-free configuration was used instead of Fabry–Pérot cavities or plasmonic nanostructures to obtain ultrastrong light–matter coupling [221]. The research explored how photochromic molecules could be used to manipulate phase singularities through strong light–matter coupling. By embedding spiropyran molecules in a polymethylmethacrylate host matrix and then converting them to merocyanine by UV exposure, the researchers have demonstrated a transition from weak to strong coupling regimes. This transition leads to the formation of pairs of phase singularities, which are regions where the phase of light is indefinite. These singularities depend on the merocyanine concentration and the film thickness, which provides a way to control them optically. This method offers a new, simple platform for phase optics and highlights the potential of using strong coupling for developing advanced photonic devices with potential applications in sensing. Phase singularities have already been used for these purposes [222,223,224]. These advanced configurations could favor plasmon biosensing. Plasmonic biosensing employs surface plasmon resonances in metal films ~50 nm in thickness where the metal surfaces are functionalized to achieve selectivity. However, many metals tend to corrode in biological solutions, which can decrease the performance and sensitivity of the device. Adding graphene to the metal film increases the sensitivity by three to four orders of magnitude because it guarantees the stability of the metal in liquid and preserves the plasmonic resonances under biofunctionalization [225].

The possibility of using PCF plasmonic sensors in various fields, including environmental monitoring, the chemical industry, the food industry, and biomedicine, is also widely explored [226]. One of the examples is the proposed plasmonic biosensor with gold and titanium dioxide immobilized on a PCF for analyzing blood composition. This device allows for the detection of red blood cells, hemoglobin, white blood cells, etc. with a maximum wavelength sensitivity of 12,400 nm/RIU [227]. Currently, numerous theoretical studies of this type of devices have been reported, but experimental studies are scarce due to the challenging fabrication.

WGM optical microcavities have been widely used as biosensors for detecting a variety of biomolecules due to their exceptional sensitivity to changes in their microenvironment. For example, an optofluidic sensing array for dynamic monitoring of protein aggregation has been proposed which consists of microdisks and Au nanoparticles, with the microdisks serving as WGM microcavities and plasmonic nanoparticles ensuring surface-enhanced Raman scattering. Tests have shown an enhancement factor of the Raman signal intensity as high as 10^7^–10^8^, ensuring an ultralow detection limit for R6G and methylene blue (10^−9^ M). Owing to real-time recording of the Raman spectra of proteins, the device seems promising for studying the pathogenesis and pathological treatment of Alzheimer’s disease [228]. Another research group employed a plasma-separating microfluidic array combining LSPR with an asymmetric Fabry–Pérot cavity [229] to detect Dengue virus by its NS1 biomarker at a concentration of 0.1–10 μg/mL in bovine blood [230]. Thus, WGM sensors can also be used to detect nanosized viruses, bacteria [231], and protein monolayers [232]; to monitor biomolecular interactions [233]; and to study many other objects and processes [156].

Finally, strong light–matter coupling can be used in nanofluidics to tune the optical properties of the system, thereby enabling the development of novel devices in the fields of nanofluidic photonics and optoelectronics [234]. In particular, the development of these devices will make it possible to further explore and understand the mechanisms of chemical reactions involved in photocatalysis and biological processes. Table 2 summarizes all the ways of combining strong light–matter coupling with micro-/nanofluidics discussed above.

## 6. Future Prospects and Concluding Remarks

The large variety of devices combining microfluidics and strong coupling presented in this review indicates their broad potential research value and future practical applications. Some of the benefits expected from using microfluidics in combination with strong coupling are reduced consumption of materials and reagents due to precise control of the sample volume, enhanced sensitivity, increased product yields, improved portability, as well as faster and less expensive techniques of the detection of chemical and biomedical entities compared with the existing approaches to handling biological samples on the macroscale. Micro-/nanofluidic architectures further amplify these benefits by providing tight fluidic confinement and precise fluidic control at the micro- and nanoscale. This precise control is ideally matched with the integrated optical characteristics of micro- and nanocavities, making the combination highly effective for various applications. Over the years, a growing number of emerging technologies have begun to be used in microfluidics, which involves next-generation materials and detection methods making it possible to develop multifunctional microfluidic chips. However, further improvements are needed to select the optimal conditions for integrating fluid flows on both micro- and nanoscales with a strong light–matter coupling regime. In this regard, different configurations have been studied in order to design this type of integrated devices.

On the one hand, Fabry–Pérot microcavities based on metal mirrors are relatively easy to fabricate and have a large surface area, suitable for spectroscopic analysis of collective strong coupling and for integrating with fluidic devices. However, they may suffer from incorrect detection of the strong coupling signal, and they may also be expensive because they require high-quality mirrors and are limited in size because they should match half the wavelength, making it especially difficult to engineer devices that operate in the visible range. Porous silicon has been proposed as a material for Fabry–Pérot microcavities based on distributed Bragg reflectors (DBRs). However, further developments are required to integrate them with fluidics because the embedment of the molecules for subsequent detection is limited by the geometry and size of the pores. Small nanopores may restrict the flow of the analyte, which leads to the formation of an unwanted layer on the top of the porous silicon microcavity. Another type of cavities, Casimir cavities, represents an approach to nanoscale separation of the reflective surfaces. However, they are at the early stages of development, and more experimental research is needed.

An alternative way to deal with subwavelengths is tip-induced light–matter interactions [149] and plasmonic cavities, which are nanoscale objects often requiring microscopy techniques to be probed; however, they offer the possibility of direct contact with the formed polaritons because the cavity is open. It should be noted that this possibility is important not only for detection but also for controlling chemical reactions. In addition, the possibility of VSC between a single nanogap antenna and several molecules is important not only from the practical point of view, due to potential applications in ultrasensitive spectroscopy, site-selective chemistry, and compact molecular polariton-based devices, but also from the theoretical one because it could help solve many-body physics problems.

PCF SPR is also an interesting option for detection applications. However, the sensors based on it are still in the early stage of development. Most studies in this field are either at the proof-of-concept level or represent theoretical and computational simulations because of the technical challenges in fabricating these devices. Therefore, further experimental research is needed to validate these concepts and demonstrate the potential future applications in the detection of analytes for a wider range of chemical and biological samples. In addition, it is necessary to deal with some problems, such as the lack of low-cost technology for fabricating metal nanostructures with a large surface area and high uniformity. In the case of LSPR sensors, the main problem is that no tests have been carried out in situ; i.e., there might be a discrepancy between the test results and the real situation. Moreover, there is a lack of reliable methods for fixing the nanoparticles.

Finally, WGM sensors are ideal for RI detection. This is a particularly attractive detection methodology because the signal increases with an increase in analyte concentration rather than volume, making it suitable for small-volume applications. One of the advantages of employing WGM and PCF SPR in fluorescent sensors is the possibility of label-free detection, which relies on the intrinsic properties of the target substance and, hence, requires fewer reagents and detection steps. In addition, these sensors can be engineered to be reusable, which helps in detecting different types of analytes. Other advantages of WGM technology are low-cost manufacturing and simple packaging techniques that protect the device from damage and that can be integrated with microfluidics. The possibility of analyzing samples in situ in real time makes these sensors suitable for rapid, portable, lab-on-a-chip assays to be used in point-of-care diagnoses. The performance could be further improved by testing several samples on a single chip. It remains an ongoing challenge to improve LOD because the target biomarkers are often contained in body fluids at trace concentrations. Additionally, noise should be reduced for the detection of smaller quantities of the analyte. This requires dealing with various sources of noise, including environmental fluctuations (related to temperature, RI, particles, etc.), mechanical vibrations, and laser noise. Since ring WGM resonators detect only a shift of the resonance wavelength but have no mechanism to distinguish all sources of the associated dielectric disturbances that could contribute to the shift, it is important to provide selectivity in order to ensure that the shift is related only to the analyte of interest. Therefore, the surface should be treated using some blocking method to make it inert to nonspecific contaminations. Point-of-care sensors should be capable of operating at the location of the patient without a permanent dedicated space or highly trained personnel. In the particular case of microbubble resonators, additional optimization is required due to the difficulty of rapid mass-scale production, using the conventional semiconductor processing technology. Integration of a tunable laser is also difficult because of the size restrictions and could compromise stability. It is important to maintain the same sensor performance outside the laboratory environment for its applicability in real-world measurements. 

The main challenges entailed with the integration of these optical devices with fluidics are the following. (1) Control and stability require careful design and fabrication of devices, as well as the development of robust control systems. (2) Integration of different technologies requires interdisciplinary collaboration and expertise, as well as the development of novel fabrication and integration methods. (3) The development of scalable fabrication methods and the optimization of device performance are needed. (4) The device parameters should be optimized, with the necessary volumes of fluids minimized and new sensitive detection methods developed. (5) Theoretical models and experimental techniques should be developed to study and manipulate fundamental interactions between light and matter in microfluidic systems. (6) Real-time control and monitoring of microfluidic devices and strong light–matter coupling systems are challenging in terms of the development of fast and precise control technologies, as well as the integration of sensing and feedback systems. (7) The cavity parameters, such as the cavity length, mirror reflectivity, and material properties, should be optimized to obtain the desired strength of light–matter coupling. This may involve iterative simulations and experimental adjustments. (8) The cavity should be installed in a controlled environment, such as a vacuum chamber or inert gas atmosphere, to minimize contamination and unwanted interactions with the surrounding medium. (9) The flatness (in the case of a nanofluidic cavity), viscosity, concentration, adsorption, and other parameters should be controlled.

Recent studies have demonstrated that confined electromagnetic fields required for the strong coupling regime can be obtained without the use of external structures (such as planar microcavities or plasmonic nanostructures). This means that electronic or vibrational excitations in bulk or nanostructured materials can self-couple to an optical mode sustained by its own geometry. This occurs because polaritonic states are natural and ubiquitous to bulk materials and nanostructures that can be described by a generic Lorentz resonance(s). Here, the boundaries of the Lorentz medium play the role of the mirrors that allow for the formation of well-defined optical modes, which, in turn, couple to the resonant transition [25,92,235,236].

VSC in a monolithic self-coupled photonic crystal operating in the terahertz range has also been reported. In this structure, the confinement is due to total internal reflection. Therefore, its Q-factor is only limited by the surface roughness and the optical out-coupling. This system is fabricated in a simpler way than DBRs (e.g., by physical or chemical vapor deposition, lithography, or etching) and could be more suitable for polaritonic chemistry because no additional external materials are used [236].

**Table 2:** Configurations that integrate the strong light–matter coupling regime with micro- and nanofluidics.

Configuration	Schematics	Principle of Operation	Pros	Limitations	Applications	Example of Use in Sensing	Ref.
Photonic crystal fiber (PCF) plasmonic sensor	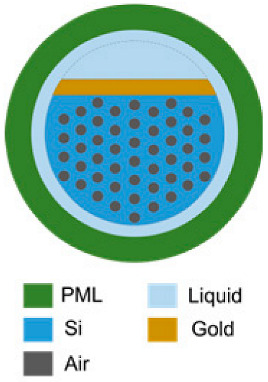	Guided light confined in a periodic dielectric structure and enhanced by plasmonic effects	High sensitivity and tunability by adjusting fiber structure; Sensitivity to refractive index changes and possibility to detect a broad spectrum of analytes; Compact size, with; very small volumes of the analyte required.	Difficulty to fabricate; most studies use simulations; Poor attachment of the bio-analyte to the sensor surface and a large distance between the core and the plasmonic layer. Potential losses due to imperfections in fiber.	Chemical and biological label-free sensing and environmental monitoring based on RI measurement.	D-shaped PCF biosensor based on a plasmonic layer with a resolution of 9.53 × 10^−6^ RIU and a maximum sensitivity of 10,493 nm/RIU.	[237]
Fabry–Pérot (F–P) microcavity	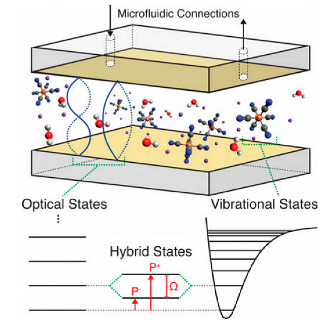 * 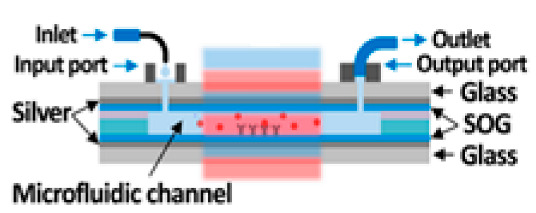 **	Multiple reflections between two parallel mirrors	Simple design; good control over resonance properties; enhanced light–matter interactions; sensitivity to refractive index changes; large surface area	Difficulty to fabricate, especially when dealing with electronic transitions. Large mode volumes; Critical alignment of mirrors required; Losses over time and limited scalability; Q-factor limited by the intrinsic reflectivity of the metals; Optical response depending on mirror roughness.	Optical filtering, spectroscopy, cavity-enhanced label sensing; Control of chemical reactions and biosensing.	LOD = 15 mM with a Rabi splitting of ~20 cm^−1^ for K_4_[Fe(CN)_6_]; LOD = 1.35 nM for streptavidin; LOD = 377 pM for human C-reactive protein.	[85,86]
Plasmon cavity	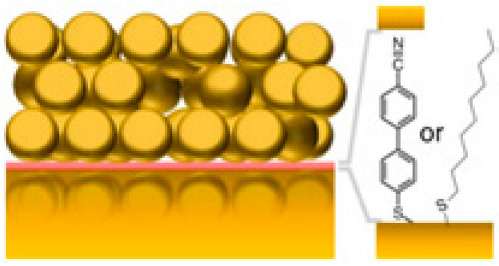 †	Localized surface plasmon resonance is used to confine light at the plasmonic resonance frequency	Extremely strong field confinement with a subwavelength effective mode volume where the diffraction limit is overcome.	Low Q-factors and high ohmic losses; Environmental instability; Precision position of molecules in nanosized plasmonic mode volumes required; Fluorescence quenching and corrosion in biological solutions.	Nanoscale label sensing and molecular detection; Ultrasensitive spectroscopy; Site-selective chemistry and compact molecular polariton-based devices; Control of chemical reactions; Engineering single-photon sources.	LOD = 100 pg/mL for poly-γ-D-glutamic acid. Coupling strength g ~ 100 cm^−1^ for molecular glue.	[238,239]
Whispering-gallery-mode (WGM) microcavities	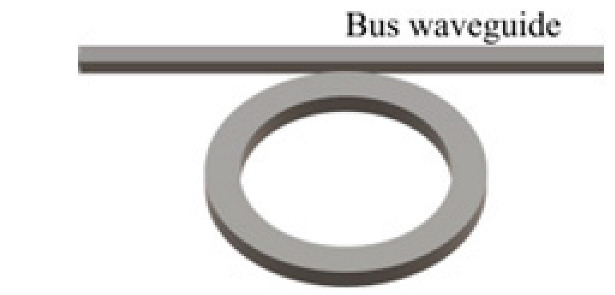 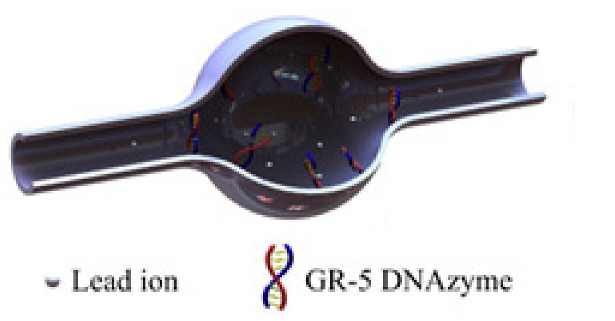 ‡	Total internal reflection at a curved surface leading to a circular light motion.	High Q-factor, minimal losses; Sensitivity to refractive index changes; High optical energy density; Versatility due to the possibility of integration of additional components, such as plasmonic nanoparticles; Low sample requirement.	Fabrication complexity; Mechanical instability	Biochemical, temperature, and mechanical label-free sensing; Single-molecule detection; Monitoring single-molecule biochemical reactions.	LOD = 2.5 ng/mL for carcinoembryonic antigen, a pancreatic cancer biomarker; LOD = 15 fM and dynamic detection range of 0.1–100 pM for GR-5 DNAzyme; LOD = 0.41 pM for D-biotins.	[177,240,241]

Reproduced with permission from * Casey and Sparks (2016) [85], published by the American Chemical Society; ** Rho and Kim (2020) [86], published by MDPI; † Arul et al. (2020) [239], published by Springer Nature; and ‡ Fu et al. (2020) [240], published by Elsevier.

## Figures and Tables

**Table 1:** Fabrication methods for the different whispering-gallery mode microcavity.

Whispering-Gallery Mode Microcavity	Scheme		Fabrication Method	Ref.
Microsphere	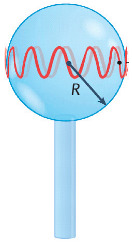	*	Heating of an optical fiber tip with CO_2_ laser, arc discharge, or H_2_ flame	[153]
Microbubble	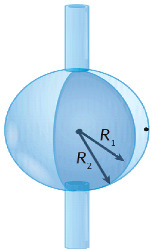	*	Local heating of a capillary with CO_2_ laser, arc discharge, or H_2_ flame, while applying internal aerostatic pressure to inflate the SiO_2_	[153]
Microbottle	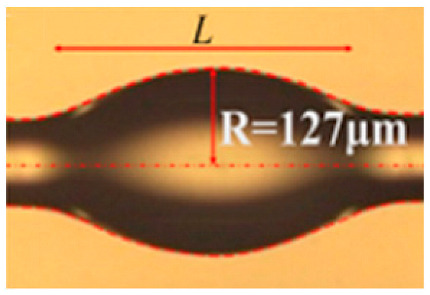	**	-Heating and stretching two regions into thinner ones -Heating and softening an optical fiber and squeezing it along the axis to build up material in the pinched region	[171]
Microdisks	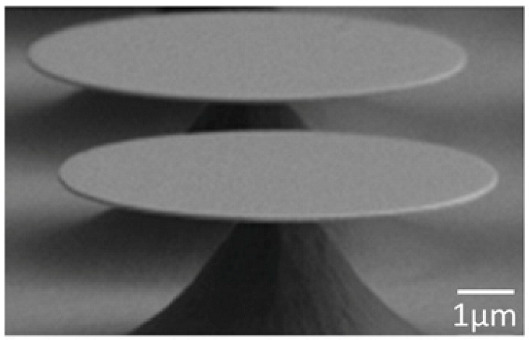	†	Lithographically forming disks by wet and dry chemical etching	[172]
Microrings	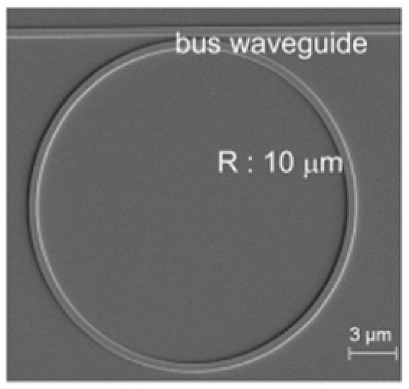	‡	Photolithography and etching, without laser reflow or other high-temperature process	[173]
Microtoroid	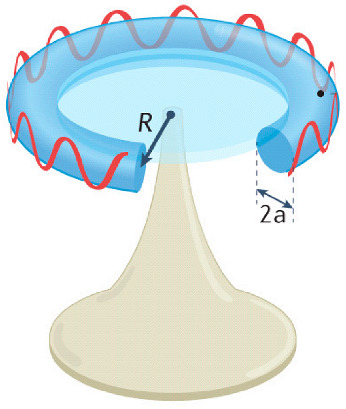	*	Photolithography to form the disk, etching with HF for the thermal oxide layer CO_2_ laser to melt the silica at the edge	[153]

Reproduced with permissions from * Yu et al. (2021) [153], published by Springer Nature; ** Zhu et al. (2020) [171], published by Elsevier; † Ghulinyan et al. (2008) [172], published by Optica (formerly known as the Optical Society of America, OSA); and ‡ Lin et al. (2010) [173], published by the American Chemical Society.

## Data Availability

Not applicable.

## Supplementary Materials

The following supporting information can be downloaded at: https://www.mdpi.com/article/10.3390/nano14181520/s1, Figure S1: Approaches to studying light–matter interaction; Table S1: Models describing the interaction between matter and electromagnetic field; Equations (S1)–(S24); the corresponding explanation text. Refs. [48,242,243,244,245,246] are cited in Supplementary Materials.
