# Supplementary material for: Microfluidics and Nanofluidics in Strong Light–Matter Coupling Systems
Source: Nanomaterials (Basel). 2024 Sep 19;14(18):1520. doi: 10.3390/nano14181520 (PMC11435064; doi:10.3390/nano14181520)
Supplement: Supplementary file 1 [file nanomaterials-14-01520-s001.zip › nanomaterials-3178160-supplementary.pdf]

Supplementary Information

Microfluidics and Nanofluidics in Strong Light–Matter Coupling Systems

Evelyn Granizo [1,2], Irina Kriukova [1,2], Pedro Escudero-Villa [3], Pavel Samokhvalov [1,2] and Igor Nabiev [1,2,4,5*]

[1] Life Improvement by Future Technologies (LIFT) Center, Skolkovo, 143025 Moscow, Russia; aleroman16@hotmail.com (E.G.); irina.kryukova.mephi@gmail.com (I.K.); p.samokhvalov@gmail.com (P.S.)
[2] Laboratory of Nano-Bioengineering, Moscow Engineering Physics Institute, National Research Nuclear University MEPhI, 115409 Moscow, Russia
[3] Facultad de Ingeniería, Universidad Nacional de Chimborazo, 060108 Riobamba, Ecuador; pedro.escudero@unach.edu.ec (P.E.)
[4] Department of Clinical Immunology and Allergology, Institute of Molecular Medicine, Sechenov First
Moscow State Medical University (Sechenov University), 119146 Moscow, Russia
[5] BioSpectroscopie Translationnelle (BioSpecT)—UR 7506, Université de Reims Champagne-Ardenne,
51100 Reims, France
* Correspondence: igor.nabiev@univ-reims.fr

A. Quantum Approach

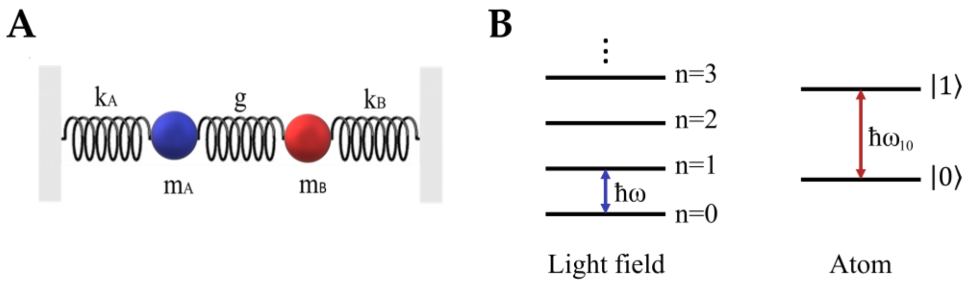

Figure S1 Approaches to studying light–matter interaction. (A) Classical approach. Both light and atom are considered as classical waves. The light-matter interaction is analogues with a system composed of two coupled oscillators which periodically transfer energy to each other. (B) Quantum approach. Both light and atom are quantized. The light-matter interaction consists in the exchange of an elementary quantum of energy (photon)

In order to understand the basics of the fully quantum-mechanical models, let us consider the simplest situation in the interaction between a quantized EM field and an atomic system, i.e., a single-mode field interacting with a two-level atom. Thus, a two-level

emitter that can only be in the ground or excited state ($|g\rangle$ and $|e\rangle$, respectively) is placed in a cavity. The full system is described by the Hamiltonian [242]

$$\hat{H} = \hat{H}_{field} + \hat{H}_{atom} + \hat{H}_{\text{int}}, \tag{S1}$$

$$\hat{H}_{field} = \hbar\omega_c \hat{a}^\dagger \hat{a}, \tag{S2}$$

$$\hat{H}_{atom} = \frac{\hbar\omega_a}{2}\hat{\sigma}_z, \tag{S3}$$

where $\hat{H}_{field}$ Hamiltonian of the electric field with resonance at $\hbar\omega_c$, $\hat{H}_{atom}$ is the Hamiltonian of the free atom with the atomic transition energy $\hbar\omega_a = E_e - E_g$, $\hat{a}^\dagger$ and $\hat{a}$ are the creation and annihilation operators of a single-mode bosonic field, which satisfy the canonical commutation relation $[\hat{a}, \hat{a}^\dagger] = 1$ and $\hat{\sigma}_z = |e\rangle\langle e| - |g\rangle\langle g|$ is the Hermitian inversion operator.

The third term of Equation (1) is the interaction Hamiltonian describing only transitions of the type $|e\rangle|n\rangle \leftrightarrow |g\rangle|n+1\rangle$, where n = 0, 1, 2, … is the photon number; i.e., one photon is emitted when the atom makes a transition from the excited state to the ground one (or one photon is absorbed, in the case of $|g\rangle \to |e\rangle$ transition). The product states of the unperturbed atom and field $|e\rangle|n\rangle, |g\rangle|n+1\rangle$ are referred to as *bare* states of the JC model. Thus, the full JC Hamiltonian for a two-level quantum system placed in a cavity takes the form

$$\hat{H}_{JC} = \hbar\omega_c \hat{a}^\dagger \hat{a} + \frac{\hbar\omega_a}{2}\hat{\sigma}_z + \hbar g(\hat{\sigma}_+ \hat{a} + \hat{\sigma}_- \hat{a}^\dagger), \tag{S4}$$

where $\hat{\sigma}_+ = |e\rangle\langle g|$ and $\hat{\sigma}_- = |g\rangle\langle e|$ are the atomic rising and lowering operators, and g is proportional to the dipole moment. The Hamiltonian has the following eigenstates:

$$|1n\rangle = \cos\theta_n |e,n\rangle + \sin\theta_n |g,n+1\rangle, \tag{S5}$$

$$|2n\rangle = -\sin\theta_n |e,n\rangle + \cos\theta_n |g,n+1\rangle, \tag{S6}$$

where $\theta_n = \frac{1}{2}\tan^{-1}\left(-\frac{2g\sqrt{n+1}}{\Delta}\right)$, for the nonresonant case $(\Delta = \omega_c - \omega_a \neq 0)$. For the resonant case $(\Delta = 0)$, the eigenstates are the following:

$$|1n\rangle = \frac{1}{\sqrt{2}}\left(|e,n\rangle + |g,n+1\rangle\right), \tag{S7}$$

$$|2n\rangle = \frac{1}{\sqrt{2}}\left(-|e,n\rangle + |g,n+1\rangle\right), \tag{S8}$$

The eigenstates (5)–(8) are the so-called *dressed* states of the atom, i.e., the eigenstates of the atom “dressed” by the cavity mode. The corresponding eigenenergies are:

$$E_{1n} = \hbar\omega_c\left(n+\frac{1}{2}\right)+\frac{1}{2}\hbar\Omega_n, \tag{S9}$$

$$E_{2n} = \hbar\omega_c\left(n+\frac{1}{2}\right)-\frac{1}{2}\hbar\Omega_n, \tag{S10}$$

where $\Omega_n = \sqrt{\Delta^2 + 4g^2(n+1)}$ is the n-photon generalized Rabi frequency. The energies of the bare levels $|e\rangle|n\rangle$ and $|g\rangle|n+1\rangle$ cross at resonance $(\Delta = 0)$, but the atom-field interaction eliminates the degeneracy, thus leading to anticrossing of the dressed states $|1n\rangle$ and $|2n\rangle$. The energy separation between the dressed states of the same manifold is $E_{1n} - E_{2n} = \hbar\Omega_n$, with the minimum value $\left|2\hbar g\sqrt{n+1}\right|$ at resonance $(\Delta = 0)$. Depending on the problem, other models of the interaction between the two-level emitter(s) and the field in the cavity, described in detail in [243–245], are considered. In the multi-emitter case, with N two-level systems interacting with the cavity field, collective two-level operators are usually introduced instead of those given above:

$$\hat{S}_x = \sum_{i=1}^{N}\hat{\sigma}_x^{(i)}, \hat{S}_\pm = \sum_{i=1}^{N}\hat{\sigma}_\pm^{(i)}, \tag{S11}$$

Table S1. Models describing the interaction between matter and electromagnetic field

Model name	Rotating wave approximation	Number of two-level systems	Interaction Hamiltonian	Schematic representation	Ref.
Quantum Rabi	NO	N=1	$\hat{H}_{\text{int}} = \hbar g(\hat{a}^\dagger + \hat{a})\hat{\sigma}_x$	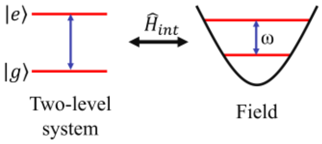	[245]
Jaynes–Cummings	YES	N=1	$\hat{H}_{\text{int}} = \hbar g(\hat{\sigma}_+\hat{a} + \hat{\sigma}_-\hat{a}^\dagger)$		[245]

Dicke	NO	N>1	$\hat{H}_{\text{int}} = \hbar g(\hat{a}^{\dagger} + \hat{a})\hat{S}_x$	* 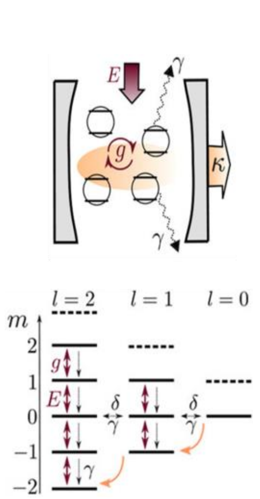	[245, 246]
Tavis–Cummings	YES	N>1	$\hat{H}_{\text{int}} = \hbar g(\hat{S}_{+}\hat{a} + \hat{S}_{-}\hat{a}^{\dagger})$		[245]

*Reproduced with permission from Gegg M., et al. (2018), published by IOP Publishing Ltd on behalf of Deutsche Physikalische Gesellschaft [246].

B. Photonic-Crystal Fiber Plasmonic Sensor

Photonic-Crystal Fiber (PCF) plasmonic sensors utilize the unique properties of photonic crystals and surface plasmon resonance (SPR) for highly sensitive analyte detection. Light dispersion within these sensors is characterized using the Sellmeier equation, while key performance metrics such as confinement loss, sensitivity, and resolution are derived from the effective refractive index and resonant wavelength shifts. These metrics, along with parameters like signal-to-noise ratio (SNR) and figure of merit (FOM), are crucial for evaluating sensor effectiveness.

In order to determine the dispersion of light in the medium, the Sellmeier equation is used:

$$n^2(\lambda) = 1 + \sum_i \frac{B_i \lambda^2}{\lambda^2 - C_i}, \tag{S12}$$

where n is the refractive index, λ is the wavelength, and B_i and C_i are experimentally determined Sellmeier coefficients.

With increasing number of air holes, the n_{eff} contrast between the core-guided mode and surface plasmon polariton mode is reduced, which results in a strong coupling field. The confinement loss is calculated from the imaginary part of the n_{eff} values of the core-guided mode:

$$\alpha_{loss}\left[\frac{dB}{cm}\right] = 8.686 \times \frac{2\pi}{\lambda} \cdot \text{Im}\left[n_{eff}\right] \times 10^4, \tag{S13}$$

where λ is the operating wavelength and $Im[n_{eff}]$ is the imaginary part of the n_{eff} of the core-guided fundamental mode.

The performance of a PCF SPR sensor is evaluated in terms of its sensitivity, i.e., the response of the sensor to the applied perturbation. The wavelength sensitivity of a PCF SPR sensor estimated by the wavelength interrogation method is

$$WS\left[\frac{nm}{RIU}\right] = \frac{\Delta\lambda_{peak}}{\Delta n_a}, \tag{S14}$$

where $\Delta\lambda_{peak}$ is the shift of the peak position and Δn_a is the change in the analyte refraction index. RIU denotes refractive index unit.

The sensor resolution indicates how good the device is in detecting a minimum variation in the analyte refractive index. It is estimated as

$$R[RIU] = \Delta n_a \times \frac{\Delta\lambda_{\min}}{\Delta\lambda_{peak}}, \tag{S15}$$

where Δn_a is the change in the analyte refraction index, $\Delta\lambda_{\min}$ is the minimum spectral resolution , and $\Delta\lambda_{peak}$ is the largest shift of the resonant wavelength.

The LOD, i.e., the smallest perturbation that can be reliably detected can be estimated as

$$LOD\left[\frac{RIU^2}{nm}\right] = \frac{R}{WS}, \tag{S16}$$

In addition to the sensitivity and sensor resolution, the signal-to-noise ratio (SNR), detection limit (δn), and figure of merits (FOM) are important parameters of the sensor performance. These parameters can be calculated by the following equations:

$$SNR = \frac{\Delta\lambda_{res}}{\Delta\lambda_{1/2}}, \tag{S17}$$

$$\delta n = \frac{\Delta\lambda_{1/2}}{1.5(SNR)^{0.25}}, \tag{S18}$$

$$FOM[RIU^{-1}] = \frac{WS}{\Delta\lambda_{1/2}}, \tag{S19}$$

where $\Delta\lambda_{res}$ is the shift in the resonant wavelength due to changes in the analyte's refractive index and $\Delta\lambda_{1/2}$ is the FWHM of the loss characteristic curve.

PCF plasmonic sensors represent a significant advancement in optical sensing technology, offering exceptional sensitivity and precision in detecting changes in analyte refractive indices. By utilizing the Sellmeier equation to understand light dispersion and calculating crucial performance metrics such as confinement loss, wavelength sensitivity, and sensor resolution, these sensors can be finely tuned for optimal performance. The introduction of additional parameters like SNR, detection limit, and FOM provides a comprehensive framework for evaluating sensor efficacy.

C. Plasmonic Nanocavity

Usually, plasmonic nanocavities are described in terms of the Jaynes–Cummings model, which provides a framework for understanding the interaction between a system such as an emitter and a nanocavity. The coupling of an emitter with an isolated nanocavity is characterized by the decay Γ_{tot} [48]:

$$\Gamma_{tot} = \Gamma_0 + \Gamma_{cav}, \tag{S20}$$

Here, Γ_0 is the decay rate of the emitter in free space, which is determined as the sum of its radiative and nonradiative decay rates and is inversely proportional to the lifetime τ_0 of the excited state of the emitter:

$$\Gamma_0 = \frac{1}{\tau_0} = \Gamma_{rad}^0 + \Gamma_{nrad}^0, \tag{S21}$$

The contribution of the nanocavity Γ_{cav} in the weak-coupling regime is calculated as follows:

$$\Gamma_{cav} = \left(\frac{4g^2}{\kappa}\right) \frac{1}{1 + \left(\frac{2\delta}{\kappa}\right)^2}, \tag{S22}$$

Where $\delta = \omega_{cav} - \omega_{em}$ is the detuning between the central frequencies of the emitter and the nanocavity. In the case of resonance, $\delta = 0$. The coupling strength is g, and the total loss rate of the cavity is $\kappa = \kappa_{rad} + \kappa_{nrad}$. Thus, the total decay rate in the strong coupling regime is

$$\Gamma_{tot} = \Gamma_0 + \frac{4g^2}{\kappa}, \tag{S23}$$

Then, the coupling strength can be obtained from the total loss κ in the plasmonic cavity and the fluorescence lifetime of the emitter before and after coupling to the cavity:

$$2g = \sqrt{(\Gamma_{tot} - \Gamma_0)\kappa}, \tag{S24}$$

By analyzing the total decay rate Γ_{tot} and its components, researchers can gain insights into both the weak and strong coupling regimes. The ability to calculate the coupling strength g from experimental parameters further enhances the applicability of this model in designing efficient plasmonic nanocavity systems.

References

242. Meystre, P.; Sargent, M. *Elements of Quantum Optics*; Springer Berlin Heidelberg: Berlin, Heidelberg, 1991; ISBN 978-3-662-11656-2.

243. Törmä, P.; Barnes, W.L. Strong Coupling between Surface Plasmon Polaritons and Emitters: A Review. *Rep. Prog. Phys.* **2015**, *78*, 013901, doi:10.1088/0034-4885/78/1/013901.

244. Larson, J.; Irish, E.K. Some Remarks on 'Superradiant' Phase Transitions in Light-Matter Systems. *J. Phys. A: Math. Theor.* **2017**, *50*, 174002, doi:10.1088/1751-8121/aa65dc.

245. Larson, J.; Mavrogordatos, T. *The Jaynes–Cummings Model and Its Descendants: Modern Research Directions*; IOP Publishing, 2021; ISBN 978-0-7503-3447-1.

246. Gegg, M.; Carmele, A.; Knorr, A.; Richter, M. Superradiant to Subradiant Phase Transition in the Open System Dicke Model: Dark State Cascades. *New J. Phys.* **2018**, *20*, 013006, doi:10.1088/1367-2630/aa9cdd.

48. Hugall, J.T.; Singh, A.; Van Hulst, N.F. Plasmonic Cavity Coupling. *ACS Photonics* **2018**, *5*, 43–53, doi:10.1021/acsphotonics.7b01139.
